# A Comparison of Spatio-Temporal Disease Mapping Approaches Including an Application to Ischaemic Heart Disease in New South Wales, Australia

**DOI:** 10.3390/ijerph14020146

**Published:** 2017-02-03

**Authors:** Craig Anderson, Louise M. Ryan

**Affiliations:** 1 School of Mathematical and Physical Sciences, University of Technology Sydney, 15 Broadway, Ultimo, NSW 2007, Australia; Louise.M.Ryan@uts.edu.au; 2 ARC Centre of Excellence for Mathematical and Statistical Frontiers (ACEMS), Parkville, VIC 3010, Australia

**Keywords:** spatio-temporal modelling, disease mapping, Bayesian, ischaemic heart disease, Moran’s I

## Abstract

The field of spatio-temporal modelling has witnessed a recent surge as a result of developments in computational power and increased data collection. These developments allow analysts to model the evolution of health outcomes in both space and time simultaneously. This paper models the trends in ischaemic heart disease (IHD) in New South Wales, Australia over an eight-year period between 2006 and 2013. A number of spatio-temporal models are considered, and we propose a novel method for determining the goodness-of-fit for these models by outlining a spatio-temporal extension of the Moran’s I statistic. We identify an overall decrease in the rates of IHD, but note that the extent of this health improvement varies across the state. In particular, we identified a number of remote areas in the north and west of the state where the risk stayed constant or even increased slightly.

## 1. Introduction

The field of spatial modelling has grown substantially over the last several decades as advances in geographic information systems (GIS) have facilitated the incorporation of spatially-indexed outcomes and/or covariates within epidemiological and environmental models. Spatial models have a vast range of applications in areas such as disease mapping [1], air pollution monitoring [2] and ecology [3]. These approaches allow more accurate modelling of the spatial pattern of the outcome of interest by accounting for the underlying spatial autocorrelation structure. It is natural to extend these approaches to identify the changes in these spatial patterns over time, with the aim of identifying either localised or global trends. Simple spatio-temporal models have existed for over 40 years (e.g., [4]), but recent developments in both computational power and the gathering and publication of vast amounts of data have led to a recent surge in the spatio-temporal literature.

The key aim of spatio-temporal modelling is to account for the evolution of outcomes in both space and time simultaneously. Cressie and Wikle [5] provide an excellent introduction to spatio-temporal modelling and outline some of the standard techniques used in this area. Spatial data takes two key forms—areal (or lattice) data and point-referenced (or geostatistical) data—and a variety of spatio-temporal models have been developed for each type of data (e.g., [6,7,8,9]).

This paper focuses on modelling ischaemic heart disease (IHD) hospital admissions in New South Wales, Australia between 2006 and 2013. IHD is responsible for around 20,000 deaths per year in Australia, and is the most common cause of death for both males and females. IHD risk has decreased substantially over the last three decades, but these decreases are not necessarily uniform across all regions. We are therefore interested in identifying any localised inequalities that may be present for IHD risk.

The diversity of the applications, data types and conceptual approaches to modelling means that the spatio-temporal modelling literature is somewhat diffuse. Therefore, a further contribution of this paper is to provide an overview of these spatio-temporal modelling approaches in order to identify similarites and differences between these existing approaches and to make recommendations for practice. We also wish to provide a quantitative comparison of the methodologies using some form of spatio-temporal goodness-of-fit test, an area which has received little attention in the literature. We therefore propose a novel use of a spatio-temporal Moran’s I statistic [10] as a method for measuring the extent of spatio-temporal autocorrelation in the residual values from the model fits.

The remainder of this paper will be structured as follows: Section 2 provides an overview of spatio-temporal modelling, including a discussion of existing methodology for analysing spatio-temporal data; Section 3 describes our IHD dataset and outlines the modelling approaches which will be carried out in our analysis and Section 4 displays the results of this analysis. Section 5 provides a summary of the paper and recommendations in practice and Section 6 outlines the key conclusions of our work.

## 2. Methodology

The spatial (and spatio-temporal) modelling literature centres around two main data types: areal (or lattice) and point-referenced (or geostatistical). Point-referenced data structures are based on the exact geographical location of an observation being recorded, generally in the form of latitude and longitude co-ordinates. This form of data is commonly used for monitoring environmental outcomes, where spatial modelling approaches can be used to characterise the nature of an environmental outcome across the entire study region based on a finite set of monitoring stations. Areal data structures are based on the study region being partitioned into a set of non-overlapping subregions known as areal units—for example, a county being divided into a set of postcode areas. Areal data are commonly used in health applications, where confidentiality issues prevent the exact geographical locations of disease cases being recorded. Instead, only the patient’s areal unit is recorded, and the data consists of an aggregated count for each individual areal unit. Thus, it is possible to conceptualise areal data as an aggregation of point-level data. Our ischaemic heart disease data is areal, and, therefore, this section will focus on discussing existing methodology for areal data. We will first outline the methodology used in a spatial context, and then show how this can be extended to account for spatio-temporal data.

### 2.1. Spatial Modelling

The aim of areal modelling is to estimate the occurence rate of an outcome (such as disease risk) in each areal unit, thus providing a set of risk estimates that cover the entire region. We may also be interested in identifying the spatial extent of covariate effects. Consider a study region A, partitioned into *n* non-overlapping areal units such that A = {$A_{1},\ldots ,A_{n}$}. A response $Y_{i}$ is observed in each areal unit, thus providing a set of response data $Y=(Y_{1},\ldots ,Y_{n})$. Area-level covariate information $X=(x_{1}^{T},\ldots ,x_{n}^{T})$ may also be available. In most cases, it is important to account for differences in population demographics across the study region, since some subregions are likely to contain a larger at-risk population. For example, areas which have a higher percentage of elderly people are likely to have higher rates of heart disease than those with a younger population, but this does not necessarily mean that there is any underlying in disease risk rate between the regions. We can account for these demographic differences by constructing a set of expected disease counts $E=(E_{1},\ldots ,E_{n})$, where $E_{i}$ is the expected number of disease cases in area *i*. These expected counts can be constructed using either internal or external standardisation based on the age and sex demographics of the population within each areal unit.

Based on these expected counts, one simple measure of disease risk is the standardised incidence ratio (SIR), which is computed for area *i* as

(1) $${\mathrm{SIR}}_{i}=\frac{Y_{i}}{E_{i}}.$$

An SIR value larger than 1 indicates that a region has a higher than expected disease risk, while an SIR less than 1 implies a lower than expected risk. The SIR provides a useful exploratory tool but has the major disadvantage that it considers each areal unit independently and does not account for any form of spatial structure in the data. Additionally, in cases where a rare disease or a small population is being studied, some areal units may have a very low expected value $E_{i}$, and the SIR would be susceptible to small random fluctuations in the response $Y_{i}$.

It is therefore more common to use a generalised linear model to estimate disease risk. Models for spatial data are based on an underlying belief that there is some form of correlation between areal units that are close to each other geographically. Given that the response is almost always based on count data, modelling is generally based on a Poisson log-linear model of the following form:

(2) $$\begin{array}{ccc} Y_{i} & ∼ & \mathrm{Poisson}(E_{i}\mu_{i})\;i=1,\ldots ,n,\\ \log(\mu_{i}) & = & x_{i}^{T}\beta +\phi_{i}. \end{array}$$

Here, $\mu_{i}$ represents the mean risk for area *i*, β is the set of coefficients relating to the covariates *X* and $\phi_{i}$ is a random effect specific to area *i*, used to account for unexplained spatial autocorrelation. The set of random effects $\phi =(\phi_{1},\ldots ,\phi_{n})$ are commonly represented by a conditional autoregressive (CAR) prior distribution. CAR priors can typically be specified via a multivariate normal distribution

(3) ϕ∼N(0,Σ),

with a covariance function Σ that reflects the spatial correlation between the random effects. Besag et al. [11] proposed the intrinsic CAR prior with covariance matrix $\Sigma =\tau^{2}{\left(D-W\right)}^{-1}$, where $\tau^{2}$ is a conditional variance parameter and *D* and *W* are matrices determined by the neighbourhood structure of the data. D is a diagonal matrix where the *i*th entry on the diagonal is equal to the number of neighbours for areal unit *i*. *W* is a neighbourhood matrix which is defined as follows:

$$w_{ii^{\prime }}=\left\{\begin{array}{cc} -1 & \mathrm{if}\;i∼i^{\prime },\\ 0, & \mathrm{otherwise}. \end{array}\right.$$

Here, $i∼i^{\prime }$ means that areas *i* and $i^{\prime }$ are neighbours. Typically, areas are defined as neighbours if they are adjacent and thus share a common border, though other specifications are also possible [12].

This specification does not have a parameter that controls the strength of the spatial correlation, and is thus inappropriate in cases with weak spatial correlation. The work of Cressie [13] and Leroux et al. [14] led to a more generalised version of the original that accounted for different strengths of spatial correlation using an additional smoothing parameter *ρ*. The covariance matrix is redefined as $\Sigma =\tau^{2}{\left(D-\rho W\right)}^{-1}$, where *ρ* controls the level of spatial correlation present, with ρ=1 corresponding exactly to the intrinsic CAR model outlined above, and ρ=0 corresponding to complete spatial independence. It is generally straightforward to estimate *ρ* from the data.

An alternative method for handling different strengths of spatial correlation is the Besag–York–Mollie (BYM) model, proposed by Besag et al. [11]. The BYM model extends the intrinsic CAR model by including a set of spatially independent random effects, *θ* such that:

(4) $$\log\left(\mu_{i}\right)=x_{i}^{T}\beta +\phi_{i}+\theta_{i},$$

where $\theta_{i}∼N\left(0,\sigma^{2}\right)$. Different strengths of spatial correlation can be accounted for by varying the relative sizes of the random effects *φ* and *θ*. The main drawback of this approach is that only the sum $\phi_{i}+\theta_{i}$ is identifiable for each region, but, nonetheless, the BYM approach remains popular within the spatial and spatio-temporal literature.

### 2.2. Spatio-Temporal Modelling

The spatial models outlined in Section 2.1 apply to data observed at a single time point, but there are many cases where data from multiple time points are available. In addition to the spatial correlations described in the previous section, these data will also have temporal correlations, with observations at consecutive time points having more in common than those further apart. There is also the possibility of a space–time interaction, given that different areas may have different temporal trends, and these trends may be more similar in areas which are closer together geographically. Consider the case where data are collected across *J* discrete time points at each of the *n* areal units. We have response data $Y=(Y_{1},\ldots ,Y_{n})$ where $Y_{i}=\left(Y_{i1},\ldots ,Y_{iJ}\right)$ is the set of *J* observations for area *i*. We consider the case where the timepoints are the same at each location, though this is not necessary for all models.

The majority of spatio-temporal models for areal data have been developed by extending the spatial model (2) as follows:

(5) $$\begin{array}{ccc} Y_{ij} & ∼ & \mathrm{Poisson}(E_{ij}\mu_{ij})\;i=1,\ldots ,n,\;j=1,\ldots ,J,\\ \log(\mu_{ij}) & = & x_{ij}^{T}\beta +s_{i}\left(\right)+u_{j}\left(\right)+v_{ij}\left(\right), \end{array}$$

where $s_{i}\left(\right)$ is a function that captures spatial correlation, $u_{j}\left(\right)$ is a function for temporal correlation and $v_{ij}\left(\right)$ is a function capturing space–time interaction. However, while this formulation is conceptually straightforward, implementation can be difficult, especially with larger datasets. The biggest challenge in such approaches is making a choice for the spatio-temporal term $v_{ij}\left(\right)$ that accurately reflects the underlying spatio-temporal risk structure, and, in particular, the possibility of different trends occurring in different regions. The remainder of this section provides an overview of the existing literature for spatio-temporal modelling. Table 1 provides a brief summary of the literature covered in this section, and, in particular, the types of spatial, temporal and spatio-temporal effects used in each method.

One of the earliest spatio-temporal models for areal data was proposed by Bernardinelli et al. [6], who suggested a model where each areal unit has a separate linear trend. Here, $s_{i}\left(\right)=\phi_{i}$, a spatial random effect, $u_{j}\left(\right)=\beta t_{j}$, a linear trend over time and $v_{ij}\left(\right)=\eta_{i}t_{j}$, the area-specific deviation from the trend. Both *φ* and *η* are modelled by a CAR prior, as outlined in Section 2.1. This approach therefore allows each areal unit to have its own intercept modelled by $\phi_{i}$ and its own slope modelled by $\left(\beta +\eta_{i}\right)t_{j}$. Although this approach is flexible in allowing each areal unit to have its own trend, it is restrictive in requiring these trends to be linear, which may not be appropriate in many applications.

An alternative approach was outlined by Waller et al. [15], who proposed a spatio-temporal extension of the BYM model (4). The authors propose fitting a separate BYM model at each time point, which is $s_{i}\left(\right),u_{j}\left(\right)=0$ and $v_{ij}\left(\right)=\theta_{i}^{\left(j\right)}+\phi_{i}^{\left(j\right)}$, where $\phi^{\left(j\right)}$ is a set of random effects at time *j* modelled by a CAR prior, and $\theta^{\left(j\right)}$ is a set of independent random effects. This formulation allows a different spatial pattern to be estimated at each time point, with the patterns being uncorrelated from one time point to the next. A similar approach is outlined by Xia and Carlin [16], who also include an age-group specific term to allow different trends for different age groups. However, these approaches make no attempt at smoothing over time, which may not be realistic in practice. One would expect some sort of temporal correlation to exist in most spatio-temporal datasets, and models should ideally account for this.

An alternative temporal extension of the BYM model is proposed by Knorr-Held and Besag [18]. They propose a model consisting of a pair of spatial random effects and a pair of temporal effects, with each pair consisting of one structured term and one unstructured term. This model has $s_{i}\left(\right)=\phi_{i}+\theta_{i}$, $u_{j}\left(\right)=\alpha_{j}+\gamma_{j}$ and $v_{ij}\left(\right)=0$, where *φ* is modelled by a CAR prior, *α* follows a first order random walk in time and (θ,γ) represent independent random effects with mean 0 and variance *ϵ*. The authors acknowledge that their model combines space and time additively and does not make provisions for cases where there is an interaction between time and space.

Knorr-Held [20] addresses this problem by extending [18] to include an additive space–time interaction term $v_{ij}\left(\right)=\delta_{ij}$. This interaction term has a covariance structure specified by the Kronecker product of the structures of the random effects which are interacting. Of particular interest is the case where the spatially correlated random effect *φ* and the temporally correlated random effect *α* interact. In this case, the spatio-temporal random effect *δ* has a specification that combines a spatial CAR model and a random walk in time. These approaches are thus able to account for spatial and temporal trends as well as potential area-specific differences in trends, but, over longer time periods, the random walk may not capture the complexity of the temporal trends.

Non-parametric smoothing approaches provide an alternative way to capture temporal trends in spatio-temporal models. One of the first such approaches was proposed by MacNab and Dean [7], who outlined a generalised additive mixed model for estimating disease risk, combining a CAR model for the spatial pattern ($s_{i}\left(\right)=\phi_{i}$) and a set of smooth functions known as B-splines [31] for the temporal trends. The authors discuss two possible formulations for the space–time interaction term: one parametric and one non-parametric. The parametric approach follows [6] by allowing spatially correlated area-specific linear deviations from the global trend by setting $v_{ij}\left(\right)=\eta_{i}t_{j}$, where *η* follows a CAR prior. The non-parametric approach models the spatio-temporal term $v_{ij}\left(\right)$ using area-specific B-splines for each region, thus allowing nonlinear area-specific deviations. The interaction term is spatially correlated in the parametric approach, but not in the non-parametric approach, where the area-specific deviations are assumed to be independent. However, the non-parametric approach allows a great deal more flexibility and may therefore provide a more realistic estimation of temporal trends, particularly in applications covering longer time periods.

MacNab and Gustafson [32] proposed a method that combined both of these appealing properties by including spatially-varying B-splines that allow the interaction term to be nonlinear but still spatially correlated. This is achieved by allowing the set of coefficients of the area-specific B-splines to follow a CAR prior, thus inducing correlation in the spline coefficients between areas which are close together. An alternative approach to modelling the space–time interaction in this model form was proposed by Torabi and Rosychuk [27], who proposed a covariance structure similar to that used in the fully-parametric form in [20]. Here, $v_{ij}\left(\right)$ is modelled with a covariance matrix given by the Kronecker product of the spatial random effects and the B-splines. These approaches are all based on using B-splines for smoothing, but more flexible smoothing approaches are available, and these could be more appropriate in certain applications.

MacNab [23] compared the B-spline smoothing approach to a number of specifications which used either smoothing splines [33] or P-splines [34]. For the B-spline model, the author had to run multiple models, each with a different number of knots, in order to select the optimal choice, while the smoothing spline and P-spline approaches allow the suitable number of knots to be selected automatically, which has obvious computational advantages. However, the author concluded that these more flexible smoothing approaches were more sensitive to the choice of hyperprior in cases where the amount of data was limited. Nonetheless, it was noted that their performance would be likely to improve for datasets with larger amounts of temporal data.

Ugarte et al. [26] use the latitudes and longitudes of the centroid of the areal units in order to apply a spatial spline smoothing approach that was originally proposed for point-referenced data [35]. This approach uses separate P-splines for the spatial and temporal terms $s_{i}\left(\right)$ and $u_{j}\left(\right)$, and then develops a spatio-temporal effect $v_{ij}\left(\right)$ by combining these P-splines via tensor products. The authors compare this approach to the CAR approach outlined in [20] using an application of brain cancer in Spain, and identify a more gradual pattern of spatial smoothing for the P-splines approach, with this approach also exhibiting narrower confidence bands than the CAR approach. However, the authors did not carry out a simulation study to compare the performances of these methods in a case where the true spatial and temporal structure is known, and it is thus unclear whether these advantages persist across a variety of applications.

An alternative non-parametric approach to areal disease mapping is proposed by Kottas et al. [24], who contend that the risk within an areal unit can be considered as the aggregation of a continuous spatial risk surface across the area. This approach cannot easily be represented in terms of the general formulation outlined above, but instead relies on modelling continuous spatial surfaces via spatial Dirichlet processes at each time point. The risk for each areal unit at each time point is modelled as the block average of this process over the required area, and temporal correlation can be induced in these spatial risk surfaces by introducing dependence on the risk surface at the previous time point. This approach provides a potential solution to some of the modelling issues inherent in the standard adjacency-based approach to areal disease mapping, such as non-standard areal unit sizes and non-constant spatial correlation, though the complexity of the design may not be suitable for data with a large temporal scale.

Bohning et al. [19] proposed modelling the disease risk via a mixture model, with the space–time interaction terms being drawn from a mixture of Poisson distributions such that $v_{it}\left(\right)=\sum_{k=1}^{K}p_{k}f\left(y_{it},\xi_{k}\right)$, where $\sum_{k=1}^{K}p_{k}=1$ are a set of mixture weights and $f(y_{it},\xi_{k})$ is the *k*th Poisson mixture component. Bohning [21] extended this approach to outline two possible approaches for constructing spatio-temporal mixtures. The first approach identifies a separate mixture model at each time period, thus meaning that at each time point there may be a different set of Poisson distributions from which the mixture is drawn. The second approach fits a single mixture model such that the same set of Poisson distributions exists across all time points, though areas can move between these mixture components at different time points. The latter method is preferred because the mixture components remain the same at all time points and thus different time points are directly comparable. This approach has benefits in terms of identifying possible clusters in the disease risk pattern, but has been designed for applications with a small number of time points, and may become computationally complex for data with a larger temporal scale.

Both [19,21] discuss methods for introducing a random temporal effect into the mixture, but there is no attempt to account for spatial or temporal correlation. This approach is therefore unlikely to successfully estimate risk in cases where underlying spatial or temporal trends exist. However, Lawson et al. [28] extended the mixture model idea from [19] by introducing a spatial term $s_{i}\left(\right)=\phi_{i}$ modelled via a CAR model and a temporal term $u_{j}\left(\right)=\alpha_{ij}$ modelled via an autoregressive model. The mixture components are a set of temporal profiles that specify a time-dependent risk structure, and these are allocated to areal units based on a set of spatially-dependent weights that are modelled via a CAR model. The authors note that care must be taken to avoid identifiability issues within the model, and also point out a potential issue with label switching.

Another alternative specification was proposed by Congdon and Southall [22], who suggest a combination of a CAR model and a set of area-specific autoregressive time series models [17] such that $s_{i}\left(\right)=\phi_{i},u_{j}\left(\right)=0,v_{ij}=\alpha_{ij}$. Here, $\alpha_{ij}=\lambda \alpha_{i(j-1)}+\epsilon$, where *λ* controls the amount of temporal correlation, and *ϵ* is random noise. This means that each value depends on the value in that areal unit at the previous time point. This structure allows each areal unit to have a different temporal trend, but there is no structured space–time interaction, and thus no inherent spatial correlation in these area-specific trends. An alternative autoregressive approach which does account for structured space–time interaction was proposed by Martinez-Beneito et al. [25]. The spatio-temporal interaction term $v_{ij}\left(\right)=\zeta_{ij}$ can be considered as a random effect with an extended CAR prior, which is based not only on the values of its neighbours at the current time point, but also the values of those neighbours at the previous time point. The term $\zeta_{ij}$ can thus be considered to be a combination of a spatial CAR model and an autoregressive time series.

Another fully-parametric approach is outlined by Rushworth et al. [30], who proposed a single set of random effects $v_{ij}\left(\right)=\zeta_{ij}$ to account for both spatial and temporal correlation. Here, the random effects at time point 1, $\zeta_{1}=\left(\zeta_{11},\ldots \zeta_{n1}\right)$ are modelled via a CAR prior with mean 0 as in (3), and then random effects at subsequent time points are modelled via a CAR prior with mean $\lambda \zeta_{\left(j-1\right)}$ such that the random effects at one time point are dependent on the value of the random effect at the previous time point. Lee and Lawson [29] propose a similar model that can simultaneously estimate disease risk and identify spatio-temporal disease clusters. This approach includes an additional piecewise constant clustering component ω such that $v_{ij}\left(\right)=\omega_{ij}+\zeta_{ij}$. This component allows areal units in different clusters to have different baseline levels of disease risk. A maximum number of clusters is defined in advance, and an indicator function is used to allocate areal units to clusters. The clustering component can vary in size from one time point to the next, and an areal unit can be in different clusters at different time points. The authors outline a number of possible specifications of $\zeta_{ij}$, and simulation results show that simply setting $\zeta_{ij}=0$ performs best in terms of accurately identifying clusters, while a specification based on that of [30] provides the most accurate risk estimation. The former approach forces risk to be constant within clusters, which is unlikely to be realistic. Therefore, it seems more likely that the latter approach will have more applications in practice.

## 3. Application

This section outlines an application of seven of the spatio-temporal models outlined above in order to illustrate the similarities and differences between each model. The seven models selected are outlined in Table 2. These models have been chosen because there is publicly available software which allows us to apply them to our dataset. The nature of the software is outlined in Table 2; six of the models were applied using the CARBayesST R package [36], of which three are from version 1.1 of the package, and three are from the current version. The seventh model was fitted in OpenBUGS using code provided on the author’s website [37]. The seven models are all fitted to the same dataset, which relates to hospitalisations due to ischaemic heart disease (IHD) in New South Wales, Australia between 2006 and 2013.

### 3.1. Data

The study region is the state of New South Wales, which is located in south-eastern Australia. New South Wales is the most populous of the eight states and territories which make up Australia, with approximately 7.6 million residents. The state is divided into 199 administrative units known as statistical local areas (SLAs), which have been partitioned based on geographical and demographic factors. One of these administrative units, Lord Howe Island, is located around 600 km off the east coast of New South Wales, and will thus be excluded from this study. The SLA populations range from a minimum population of 752 to a maximum population of 145,865, with a median SLA population of 24,411.5. Almost two-thirds of New South Wales’ population lives in the Sydney metropolitan area, and this is reflected in the SLA structure: SLAs in the Sydney region have small areas and large populations, while more remote SLAs have very large areas but small populations.

The disease data are monthly counts of emergency hospitalisations due to ischaemic heart disease (IHD) in each SLA between January 2006 and June 2013. The data were provided by New South Wales Health, and correspond to the International Classification of Disease 10th revision codes I20–I25. The dataset has n=198 areal units, with observations taken at J=90 time points. We also have covariates relating to the patients’ age and gender, which are used to construct expected counts via internal standardisation. Population data and shapefiles were obtained from the Australian Bureau of Statistics (ABS).

**Figure 1:**
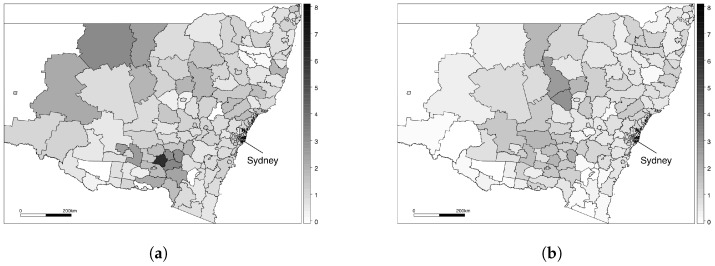
Standardised incidence ratios ($Y_{i}/E_{i}$) for statistical local areas in New South Wales. (**a**) Standardised incidence ratios for January 2006; (**b**) Standardised incidence ratios for January 2013.

In Figure 1, we display the standardised incidence ratios (1) for the first and last January of the study; note that January 2013 rather than June 2013 has been chosen in order to allow a direct month-on-month comparison. Figure 1a displays a map of the Standardised Incidence Ratios (SIRs) in January 2006, and shows that there are substantial spatial variations in ischaemic heart disease risk, with the risk appearing to be higher in the western and northern parts of New South Wales. Figure 1b shows a map of the SIRs in January 2013, and it is apparent that the disease risk has decreased overall during the study period. However, it appears that these decreases are not uniform across the region, and it appears that the risk is decreasing at a faster rate in the area around Sydney than in the more remote regions.

### 3.2. Model Comparison

Models 1–7 were applied to the data using the software outlined in Table 2, with a month of the year effect included as the only covariate in each model. Each model was fitted using Markov-Chain Monte Carlo (MCMC), with 10,000 iterations. In each case, the first 5000 iterations were discarded as burn-in, and the remaining 5000 were used to obtain our parameter estimates. The models were compared in terms of three key criteria: computing time, residual autocorrelation and model fit.

The residual autocorrelation was measured using a spatio-temporal extension of the Moran’s I statistic [38]. A spatio-temporal extension of this statistic was originally proposed as a descriptive tool for spatio-temporal data [10], but here we propose a novel use of this statistic as a goodness-of-fit test. Suppose our variable of interest *Y* is measured for *n* areal units at *J* timepoints. Then, the spatio-temporal Moran’s I statistic, MoranST, is given by

$$\mathrm{MoranST}=\frac{nJ\sum_{i=1}^{n}\sum_{j=1}^{J}\sum_{k=1}^{n}\sum_{l=1}^{J}{\tilde{w}}_{\left(ij,kl\right)}\left(Y_{ij}-\bar{Y}\right)\left(Y_{kl}-\bar{Y}\right)}{\sum_{i=1}^{n}\sum_{j=1}^{J}\sum_{k=1}^{n}\sum_{l=1}^{J}{\tilde{w}}_{\left(ij,kl\right)}\sum_{i=1}^{n}\sum_{j=1}^{J}{\left(Y_{ij}-\bar{Y}\right)}^{2}}.$$

Here, $\bar{Y}$ is the mean of the observed values $Y_{i}j$ and ${\tilde{w}}_{\left(ij),(kl\right)}$ is a weight which accounts for the spatio-temporal autocorrelation between $y_{ij}$ and $y_{kl}$, defined as:

$${\tilde{w}}_{\left(ij),(kl\right)}=\left\{\begin{array}{cc} w_{ik}, & \;ifj=l,\\ 1, & \;ifi=k\;\mathrm{and}\;|j-l|=1,\\ \;0, & \;\mathrm{otherwise}, \end{array}\right.$$

where $w_{ik}$ is a spatial weight which is equal to 1 if areas *i* and *k* are neighbours, and 0 otherwise.

This corresponds to a combination of the standard spatial neighbourhood matrix and a temporal autoregressive model with time lag 1. Any pair of observations which are either neighbours geographically and measured at the same timepoint, or measured at the same location one time point apart are considered to be “spatio-temporal neighbours” and are given a weight of 1. All other pairs of observations are given a weight of 0. A MoranST close to 1 corresponds to strong positive spatial autocorrelation, while a MoranST of 0 corresponds to complete spatial randomness.

We can adopt a similar technique to that used in spatial modelling, where the MoranST is used to test for spatial correlation in the model residuals, *e*. The residuals of a model are defined as the difference between the observed and fitted values, and can be computed as $e_{ij}=Y_{ij}-{\tilde{Y}}_{ij}$, where ${\tilde{Y}}_{ij}$ is the fitted value from the model for area *i* at time *j*. A good spatio-temporal model should account for all of the spatio-temporal trends in the data, and, in such cases, one would not expect any spatio-temporal correlation in the residuals. It is therefore possible to measure the quality of the model by computing the MoranST for the residuals; the best model is the one with the MoranST closest to zero.

The quality of the model fit is tested using the Deviance Information Criterion (DIC), which is defined as DIC = $\bar{D}+p_{d}$, where $\bar{D}$ is the mean posterior deviance and $p_{d}$ is the effective number of parameters [39]. The posterior deviance would be minimised by the best fitting model (i.e., the most complex), but this does not take into account the complexity of the model, hence the addition of the effective number of parameters as a trade off. The model with the lowest DIC can be considered to provide the closest fit to the observed data.

## 4. Results

Table 3 displays the computational time, MoranST and DIC results for fitting each of the seven selected models to our dataset. We can see that Models 1–3 and Model 6 can all fit the data in less than 200 s, while Models 4 and 5 take slightly longer, at 796 and 1178 s respectively. Model 7, the only model which was fitted using OpenBUGS rather than R, takes vastly longer to fit with our data: 14 h, compared to a maximum of 20 min for any of the other models. This increase in computing time may be prohibitive for some users, especially when working with datasets larger than this one. However, much of the time difference is likely down to the software implementation rather than the complexity of the model.

The MoranST scores for the residuals show that most of the models have done a reasonable job of accounting for the spatio-temporal trend in the dataset. The exception is Model 4, where a MoranST of 0.30 was obtained, which implies that there is still substantial spatio-temporal autocorrelation in the residuals after fitting the model. Model 5 has the MoranST score which is closest to zero—the value of −0.0066 implies that there is almost no spatio-temporal autocorrelation present in the residuals, which means the model has accounted for the trends in the data. Models 6 and 7 also perform well on this measure, with MoranST scores of −0.0092 and −0.0074, respectively.

Models 5, 6 and 7 are also the ones which perform best in terms of the DIC. Model 7 has the lowest DIC with a value of 111,032, which implies that it provides the best fit to the observed data. Model 5 has the next best DIC (112,341), followed by Model 6, which has a DIC of 112,523. Based on these results, it appears clear that Models 5, 6 and 7 represent the three most promising approaches for our data. Model 7 has the best DIC score, but it requires a substantial undertaking in terms of computing time and therefore may not be an efficient choice in practice. With that in mind, Model 5 appears to be the best choice overall. It produces the best MoranST score and the next best DIC, and although it takes a little longer to fit than some of the other models, it was still able to handle our dataset within a reasonable timescale.

**Figure 2:**
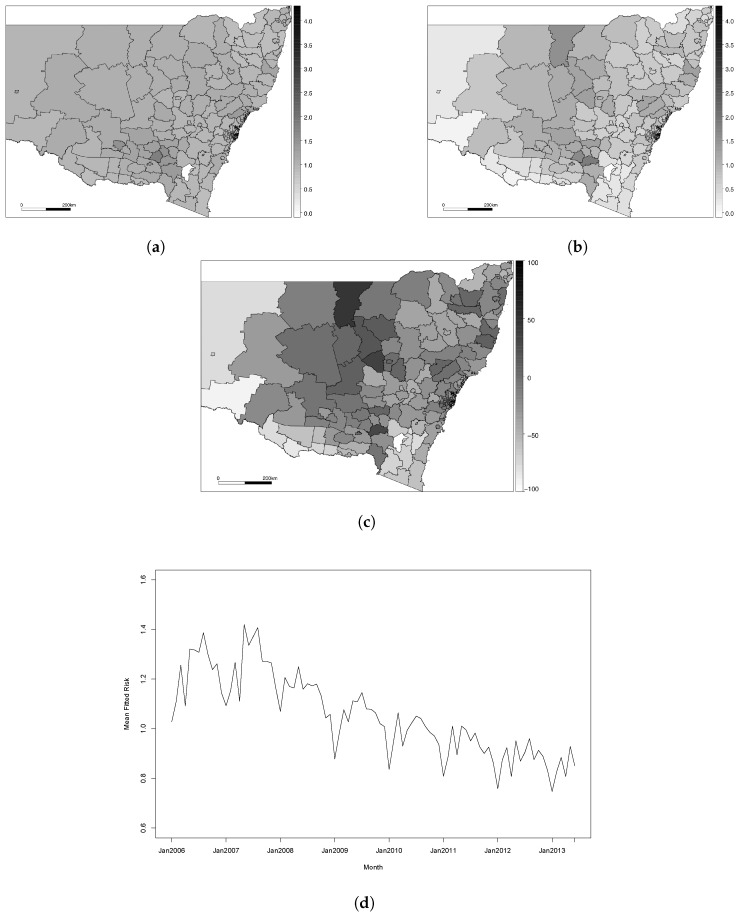
Results for Model 5. (**a**) Fitted IHD risks for January 2006; (**b**) Fitted IHD risks for January 2013; (**c**) Overall percentage change in fitted risks between January 2006 and January 2013; (**d**) Mean IHD risk in New South Wales by month.

Figure 2 displays the results of fitting Model 5 to our dataset. Figure 2a,b display the fitted values for each areal unit in the first and last January of the study period (January 2006 and January 2013, respectively), Figure 2c displays the percentage change in disease risk from January 2006 to January 2013 and Figure 2d displays the overall disease risk for the state of NSW in each month, computed as the mean of the fitted risks in each subregion. We can see from Figure 2a that the disease levels are fairly constant across the region in January 2006, but Figure 2b shows that, by January 2013, there appears to be a more pronounced spatial pattern. This is reflected in the variances of the relative risks—in January 2006, the state-wide variance was 0.027, but, by January 2013, this increased to 0.077.

In January 2013, the relative risk of disease appears to be higher in the more remote regions further away from Sydney, although this pattern is not present in the border regions where edge effects may occur due to patients travelling interstate for treatment. This is also reflected in Figure 2c, which shows small increases in fitted risk in these remote areas, while the areas closer to the city are experiencing a decrease in risk. Figure 2d shows that the risk fluctuates across the year, with higher incidence of IHD in the winter months (June–August in Australia) and lower incidences in the summer (December–February). The average risk of IHD in New South Wales has decreased over the study period. The mean relative risk in January 2006 was 1.03, while the risk in January 2013 was 0.75. This corresponds to a mean decrease in risk of 28%, but it appears that these decreases have not been uniform across the state. The overall decrease in IHD risk in the state is a result of increased awareness and advancements in treatment and diagnosis, but these effects are less pronounced in remote areas where people do not have the same level of access to health care.

**Figure 3:**
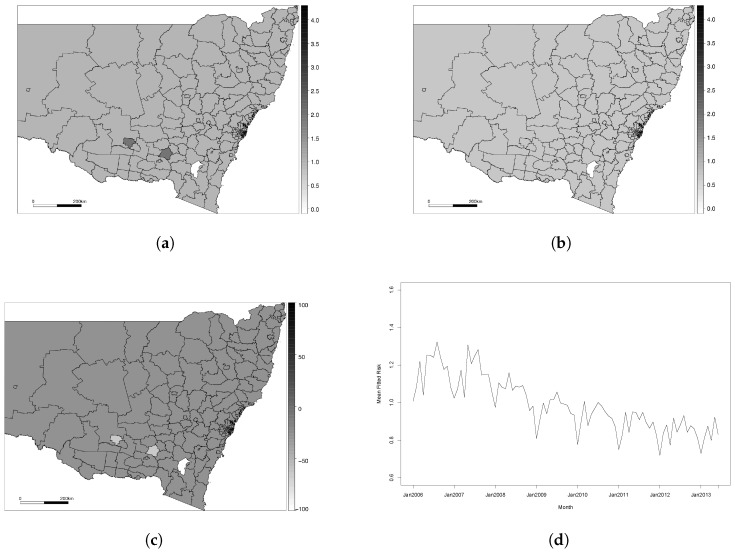
Results for Model 4. (**a**) Fitted IHD risks for January 2006; (**b**) Fitted IHD risks for January 2013; (**c**) Overall percentage change in fitted risks between January 2006 and January 2013; (**d**) Mean IHD risk in New South Wales by month.

For the purposes of comparison, and to show the benefit of a good model choice, we have included a plot of the results for Model 4 in Figure 3. Similar plots for each of the other five models are included in the Supplementary Materials (Figures S1–S7, Tables S1 and S2). Model 4 was shown to be the poorest performing model and failed to capture the spatio-temporal trend in our dataset. It should be noted that Models 4 and 5 come from the same paper [29], which proposes an underlying clustering model, and outlines two different specifications for the spatio-temporal term. Model 4 proposes a piecewise constant spatio-temporal term for each cluster, while Model 5 allows for smoothing within a cluster. It is apparent from Figure 3 that almost every element was classified in the same cluster for Model 4, which led to a poor fit for the disease risk. The specification used in Model 5 allowed for much more spatial heterogeneity in the risk trends, which led to much better estimation of the risk pattern.

**Figure 4:**
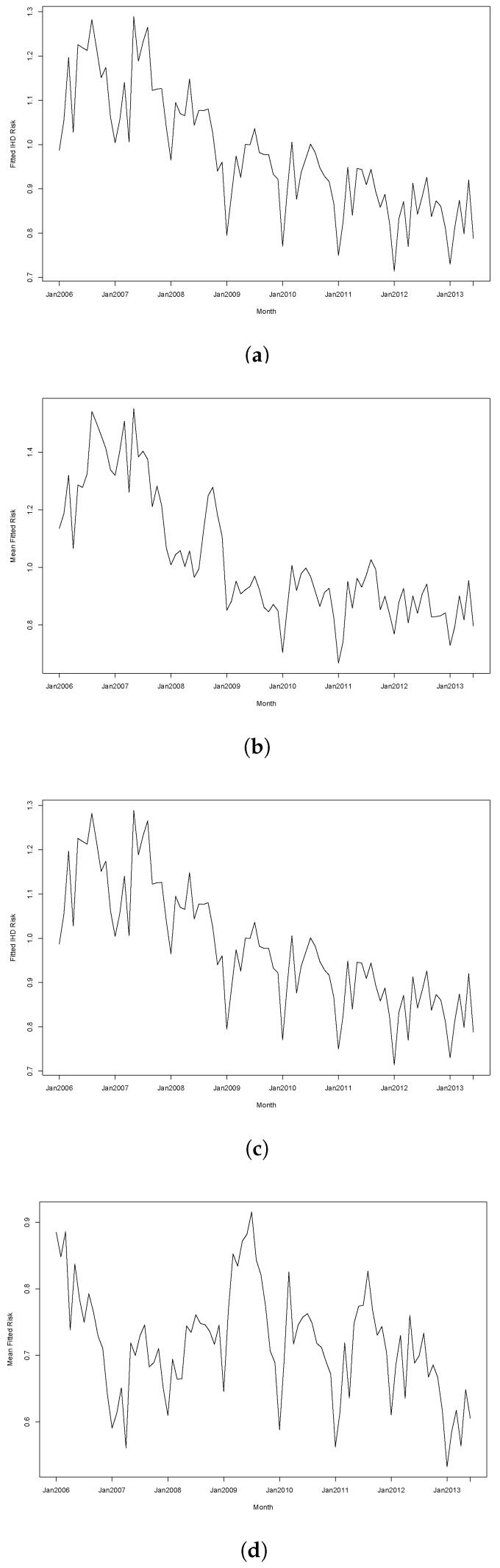
Fitted IHD trends for two randomly selected subregions using Models 4 and 5. (**a**) Trend for Lake Macquarie West using Model 4; (**b**) Trend for Lake Macquarie West using Model 5; (**c**) Trend for Uralla using Model 4; (**d**) Trend for Uralla using Model 5.

Figure 4 further illustrates the differences between these models by comparing the fitted trends for two randomly selected subregions under each model. The selected areas were Lake Macquarie West, part of a coastal city around 150 km north of Sydney, and Uralla, an inland rural town about 450 km northwest of Sydney. Figure 4a,c show that both regions have identical trends under Model 4 due to them being in the same cluster, while Figure 4b,d show that Model 5 provides more flexibility to fit individual trends for these regions.

## 5. Discussion

Spatio-temporal models are used for a wide range of applications and data types, and, consequently, the literature is quite diffuse. Section 2.2 provides an overview of these models and outlines similarities and differences between existing models. We have shown that these spatio-temporal models can be broken down into spatial, temporal and spatio-temporal terms, and we have identified the different structures used for these terms within different modelling frameworks. The early spatio-temporal literature tended to favour simpler models, often omitting a spatio-temporal interaction term, but we have identified that such a term can be useful in cases where the temporal trends in disease risk vary from region to region. Recent developments in computing power have allowed more and more complex spatio-temporal models to be fitted, and many contemporary approaches can account for such spatio-temporal differences.

In Section 3, we compared seven spatio-temporal models by applying them to data for ischaemic heart disease (IHD) hospital admissions in New South Wales, Australia. This application showed that the model proposed by Lee and Lawson [29] performed best in terms of accounting for the spatio-temporal trends present in the data, as measured by the MoranST score. The model proposed by Martinez-Beneito et al. [25] provides a slightly better fit in terms of DIC, but a major limitation of this method is the computing time required for the existing software for the approach. Based on these results, we selected the Lee and Lawson [29] approach using within-cluster smoothing as the best model for our dataset.

## 6. Conclusions

The spatio-temporal term in the Lee and Lawson [29] model allowed us to accurately capture the variation in the temporal trends across the region. The clustering structure present in this algorithm means that extreme risks are not necessarily smoothed towards the global mean, an advantage in cases where one would like to identify high (or low) risk areas. We identified that IHD risk in New South Wales decreased by around 28% between 2006 and 2013, and that disease risk was higher in the winter months than the summer months. We also identified that the decrease in IHD risk is not uniform across the state; for many remote areas in the north and west of the state, the risk stayed constant or even increased slightly. People in these remote areas have less access to healthcare than those in urban areas, and therefore have not experienced the same benefits of increased awareness and improved treatment and diagnosis of IHD.

The field of spatial modelling has grown substantially over the last couple of decades, aided by recent developments in computational power and the collection and publication of larger and more complex datasets. In particular, applications in epidemiology, ecology and meteorology have become increasingly common because of the importance of there being model changes in spatial patterns over time to identify trends. The increasing shift towards ‘big data’ has clear implications in spatio-temporal modelling, and it seems likely that the development of parallelisable spatio-temporal models will be the next key area of development.

## Figures and Tables

**Table 1:** Summary of the structures of the spatio-temporal models discussed in this paper. The following abbreviations are used in the table: AR = autoregressive model [17], BYM = Besag–York–Mollie model (4), CAR = conditional autoregressive model (3).

Paper	Space	Time	Space–Time
Bernardinelli et al. (1995) [6]	CAR	Linear	CAR
Waller et al. (1997) [15]	-	-	BYM
Xia and Carlin (1997) [16]	-	-	BYM
Knorr-Held and Besag (1998) [18]	BYM	BYM	-
Bohning et al. (2000) [19]	-	-	Poisson Mixture
Knorr-Held (2000) [20]	BYM	BYM	Kronecker
MacNab and Dean (2001) [7]	CAR	B-Spline	CAR/B-Spline
Bohning (2003) [21]	-	-	Poisson Mixture
Congdon and Southall (2005) [22]	CAR		Independent AR
MacNab (2007) [23]	CAR	B-Spline	CAR + B-Spline
Kottas et al. (2008) [24]	Dirichlet	AR	-
Martinez-Beneito et al. (2008) [25]	CAR	AR	CAR + AR
Ugarte et al. (2010) [26]	P-Spline	P-Spline	P-Spline
Torabi and Rosychuk [27]	CAR	B-Spline	Kronecker
Lawson et al. (2012) [28]	CAR	AR	Poisson Mixture
Lee and Lawson (2014) [29] (Method A)	-	-	Piecewise constant
Lee and Lawson (2014) [29] (Method B)	-	-	CAR + AR
Rushworth et al. (2014) [30]	-	-	CAR + AR

**Table 2:** Outline of the seven methods compared in this paper and the software used to fit them.

Model	Paper	Software
Model 1	Bernardinelli et al. (1995) [6]	CARBayesST
Model 2	Knorr-Held and Besag (1998) [18]	CARBayesST (v1.1)
Model 3	Knorr-Held (2000) [20]	CARBayesST
Model 4	Lee and Lawson (2014) [29] (Method A)	CARBayesST (v1.1)
Model 5	Lee and Lawson (2014) [29] (Method B)	CARBayesST (v1.1)
Model 6	Rushworth et al. (2014) [30]	CARBayesST
Model 7	Martinez-Beneito et al. (2008) [25]	BUGS

**Table 3:** Comparison of the performance of our seven models.

Model	Time (s)	MoranST	DIC
Model 1	138.6	0.0833	119,365
Model 2	189.0	0.1049	123,305
Model 3	169.5	0.0864	114,241
Model 4	795.8	0.3028	159,125
Model 5	1177.9	−0.0066	112,341
Model 6	184.3	−0.0092	112,523
Model 7	49,720.0	−0.0074	111,032

## Supplementary Materials

The following are available online at www.mdpi.com/1660-4601/14/2/146/s1. Figure S1: Results for Model 1, Figure S2: Results for Model 2, Figure S3: Results for Model 3, Figure S4: Results for Model 4, Figure S5: Results for Model 5, Figure S6: Results for Model 6, Figure S7: Results for Model 7, Table S1: Outline of the seven methods compared in this paper and the software used to fit them, Table S2: Comparison of the performance of our seven models.

## References

[1] Best N., Richardson S., Thomson A. (2005). A comparison of Bayesian spatial models for disease mapping. Stat. Methods Med. Res..

[2] Lee D., Shaddick G. (2010). Spatial modeling of air pollution in studies of its short-term health effects. Biometrics.

[3] Ver Hoef J., Peterson E., Theobald D. (2006). Spatial statistical models that use flow and stream distance. Environ. Ecol. Stat..

[4] Rodriguez-Iturbe I., Mejia J. (1974). The Design of Rainfall Networks in Time and Space. Water Resour. Res..

[5] Cressie N., Wikle C. (2011). Statistics for Spatio-Temporal Data.

[6] Bernardinelli L., Clayton D., Pascutto C., Montomoli C., Ghislandi M., Songini M. (1995). Bayesian analysis of space–time variation in disease risk. Stat. Med..

[7] MacNab Y., Dean C.B. (2001). Autoregressive Spatial Smoothing and Temporal Spline Smoothing for Mapping Rates. Biometrics.

[8] Huang H., Cressie N., Gabrosek J. (2002). Fast, Resolution-Consistent Spatial Prediction of Global Processes from Satellite Data. J. Comput. Graph. Stat..

[9] Kammann E., Wand M. (2003). Geoadditive models. J. R. Stat. Soc. Ser. C Appl. Stat..

[10] Chen S.-K., Wei W., Mao B.-H., Guan W. (2013). Analysis on urban traffic status based on improved spatio-temporal Moran’s I. Acta Phys. Sin..

[11] Besag J., York J., Mollié A. (1991). Bayesian image restoration, with two applications in spatial statistics. Ann. Inst. Stat. Math..

[12] Earnest A., Morgan G., Mengersen K., Ryan L., Summerhayes R., Beard J. (2007). Evaluating the effect of neighbourhood weight matrices on smoothing properties of Conditional Autoregressive (CAR) models. Int. J. Health Geogr..

[13] Cressie N. (1993). Statistics for Spatial Data, revised edition ed..

[14] Leroux B., Lei X., Breslow N., Halloran M., Berry D. (1999). Statistical Models in Epidemiology, the Environment and Clinical Trials. Estimation of Disease Rates in Small Areas: A New Mixed Model for Spatial Dependence.

[15] Waller L., Carlin B., Xia H., Gelfand A. (1997). Hierarchical Spatiotemporal Mapping of Disease Rates. J. Am. Stat. Assoc..

[16] Xia H., Carlin B. (1997). Spatio-Temporal Models with Errors in Covariates: Mapping Ohio Lung Cancer Mortality. Stat. Med..

[17] Chib S. (1993). Bayes regression with autoregressive errors: A Gibbs sampling approach. J. Econom..

[18] Knorr-Held L., Besag J. (1998). Modelling risk from a disease in space and time. Stat. Med..

[19] Bohning D., Dietz E., Schlattmann P. (2000). Space–time mixture modelling of public health data. Stat. Med..

[20] Knorr-Held L. (2000). Bayesian modelling of inseparable space–time variation in disease risk. Stat. Med..

[21] Bohning D. (2003). Empirical Bayes estimators and non-parametric mixture models for space and timespace disease mapping and surveillance. Environmetrics.

[22] Congdon P., Southall H. (2005). Trends in inequality in infant mortality in the north of England, 1921 to 1973, and their association with urban and social structure. J. R. Stat. Soc. Ser. A.

[23] MacNab Y. (2007). Spline smoothing in Bayesian disease mapping. Environmetrics.

[24] Kottas A., Duan J., Gelfand A. (2008). Modeling disease incidence data with spatial and spatio-temporal Dirichlet process mixtures. Biometr. J..

[25] Martinez-Beneito M., Lopez-Quilez A., Botella-Rocamora P. (2008). An autoregressive approach to spatio-temporal disease mapping. Stat. Med..

[26] Ugarte M.D., Goicoa T., Militino A.F. (2010). Spatio-temporal modelling of mortality risks using penalized splines. Environmetrics.

[27] Torabi M., Rosychuk R. (2011). Spatio-temporal modelling using B-spline for disease mapping: Analysis of childhood cancer trends. J. Appl. Stat..

[28] Lawson A., Choi J., Cai B., Hossain M., Kirby R., Liu J. (2012). Bayesian 2-stage space time mixture modeling with spatial misalignment of the exposure in small area health data. J. Agric. Biol. Environ. Stat..

[29] Lee D., Lawson A. (2014). Cluster detection and risk estimation for spatio-temporal health data. arXiv.

[30] Rushworth A., Lee D., Mitchell R. (2014). A spatio-temporal model for estimating the long-term effects of air pollution on respiratory hospital admissions in Greater London. Spat. Spatio-Temporal Epidemiol..

[31] De Boor C. (1972). On Calculation With B-splines. J. Approx. Theory.

[32] MacNab Y., Gustafson P. (2007). Regression B-spline smoothing in Bayesian disease mapping: With an application to patient safety surveillance. Stat. Med..

[33] Green P., Silverman B. (1994). Nonparametric Regression and Generalized Linear Models.

[34] Eilers P., Marx B. (1996). Flexible smoothing with B-splines and penalties. Stat. Sci..

[35] Lee D., Durban M. (2011). P-spline ANOVA-type interaction models for spatio-temporal smoothing. Stat. Model..

[36] Lee D., Rushworth A. (2014). CARBayesST: Poisson Log-Linear Models with Spatio-Temporal Random Effects. R Package. https://github.com/barryrowlingson/CARBayesST.

[37] Martinez-Beneito M. BUGS Code for AR1 Model. http://www.uv.es/mamtnez/AR1.txt.

[38] Moran P. (1950). Notes on Continuous Stochastic Phenomena. Biometrika.

[39] Spiegelhalter D., Best N., Carlin B., Van der Linde A. (2002). Bayesian measures of model complexity and fit. J. R. Stat. Soc. Ser. B.

