# Supplementary material for: A Comparison of Spatio-Temporal Disease Mapping Approaches Including an Application to Ischaemic Heart Disease in New South Wales, Australia
Source: Int J Environ Res Public Health. 2017 Feb 3;14(2):146. doi: 10.3390/ijerph14020146 (PMC5334700; doi:10.3390/ijerph14020146)
Supplement: Supplementary file 1 [file ijerph-14-00146-s001.zip › Supplementary/Supplementary.pdf]

Supplementary Materials: A Comparison of Spatio-Temporal Disease Mapping Approaches Including an Application to Ischaemic Heart Disease in New South Wales, Australia

Craig Anderson and Louise M. Ryan

1. Introduction

This document is supplementary material accompanying the manuscript titled "*A comparison of spatio-temporal disease mapping approaches including an application to ischaemic heart disease in New South Wales, Australia.*". In the manuscript, we applied seven different spatio-temporal models to data for ischaemic heart disease (IHD) hospital admissions in New South Wales, Australia. In the interests of brevity, we only included plots for two of those applications; those which performed best and worst of the seven. This document contains the relevant plots for all seven models.

2. Comparison of Models

Table S1 outlines the seven models which were applied to the IHD data, as well as the statistical software used. Table S2 compares the performance of the models in terms of computing time, MoranST and Deviance Information Criteria (DIC). These metrics are explained in further detail in the main paper.

Figures S1–S7 display the results for applying models 1–7 to our dataset. In each case, the top left and top right panels display the fitted ischaemic heart disease (IHD) risk for each areal unit in the first and last January of the study period (January 2006 and January 2013 respectively). The bottom left panel displays the percentage change in disease risk from January 2006 to January 2013 and the bottom right panel displays the mean monthly fitted values across the region.

Table S1. Outline of the seven methods compared in this paper and the software used to fit them.

Model	Paper	Software
Model 1	Bernardinelli et al. (1995)	CARBayesST
Model 2	Knorr-Held and Besag (1998)	CARBayesST (v1.1)
Model 3	Knorr-Held (2000)	CARBayesST
Model 4	Lee and Lawson (2014) (Method A)	CARBayesST (v1.1)
Model 5	Lee and Lawson (2014) (Method B)	CARBayesST (v1.1)
Model 6	Rushworth et al. (2014)	CARBayesST
Model 7	Martinez-Beneito et al. (2008)	BUGS

Table S2. Comparison of the performance of our seven models.

Model	Time (Seconds)	MoranST	DIC
Model 1	138.6	0.0833	119,365
Model 2	189.0	0.1049	123,305
Model 3	169.5	0.0864	114,241
Model 4	795.8	0.3028	159,125
Model 5	1177.9	−0.0066	112,341
Model 6	184.3	−0.0092	112,523
Model 7	49,720.0	−0.0074	111,032

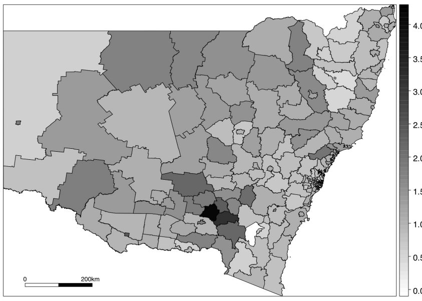

(**a**)

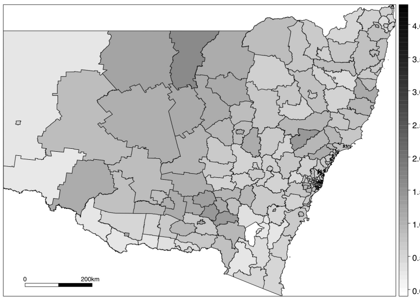

(**b**)

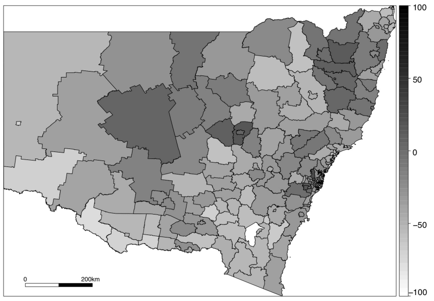

(**c**)

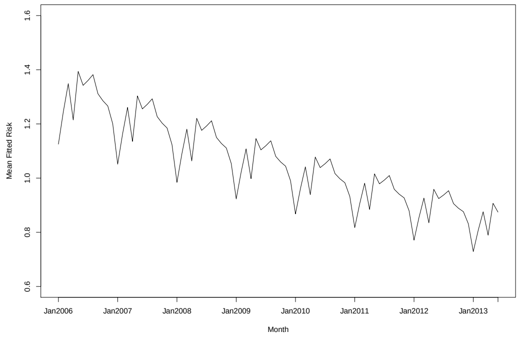

(**d**)

Figure S1. Results for Model 1. (**a**) Fitted IHD risks for January 2006. (**b**) Fitted IHD risks for January 2013. (**c**) Overall percentage change in fitted risks between January 2006 and January 2013. (**d**) Mean IHD risk in New South Wales by month.

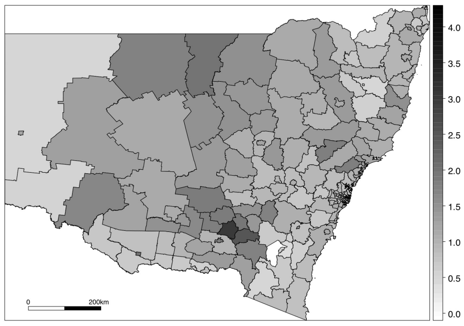

(**a**)

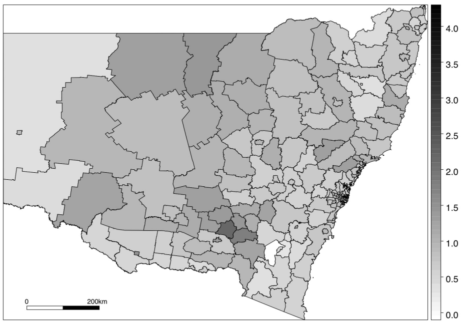

(**b**)

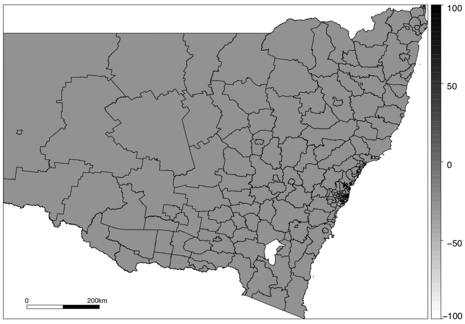

(**c**)

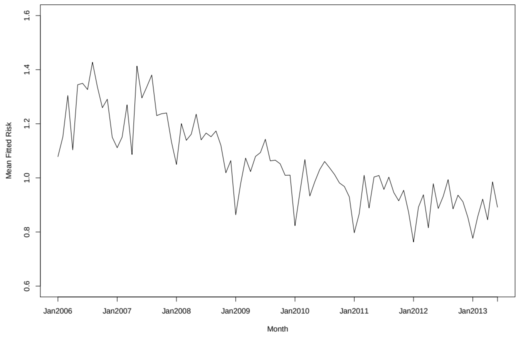

(**d**)

Figure S2. Results for Model 2. (**a**) Fitted IHD risks for January 2006. (**b**) Fitted IHD risks for January 2013. (**c**) Overall percentage change in fitted risks between January 2006 and January 2013. (**d**) Mean IHD risk in New South Wales by month.

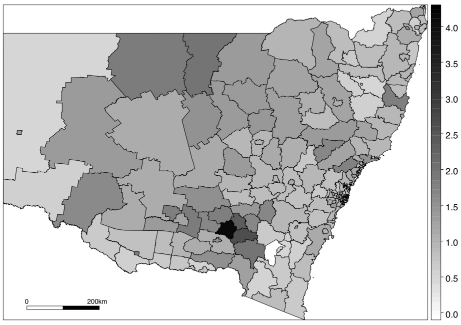

(**a**)

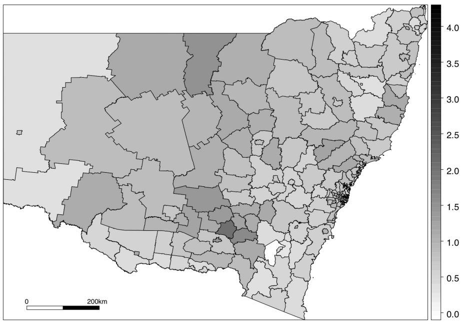

(**b**)

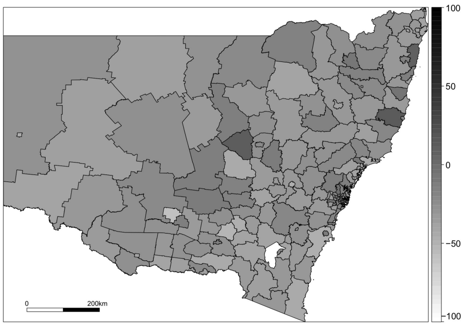

(**c**)

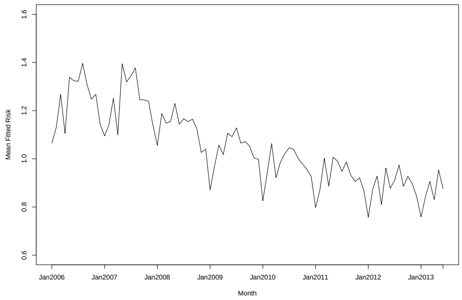

(**d**)

Figure S3. Results for Model 3. (**a**) Fitted IHD risks for January 2006. (**b**) Fitted IHD risks for January 2013. (**c**) Overall percentage change in fitted risks between January 2006 and January 2013. (**d**) Mean IHD risk in New South Wales by month.

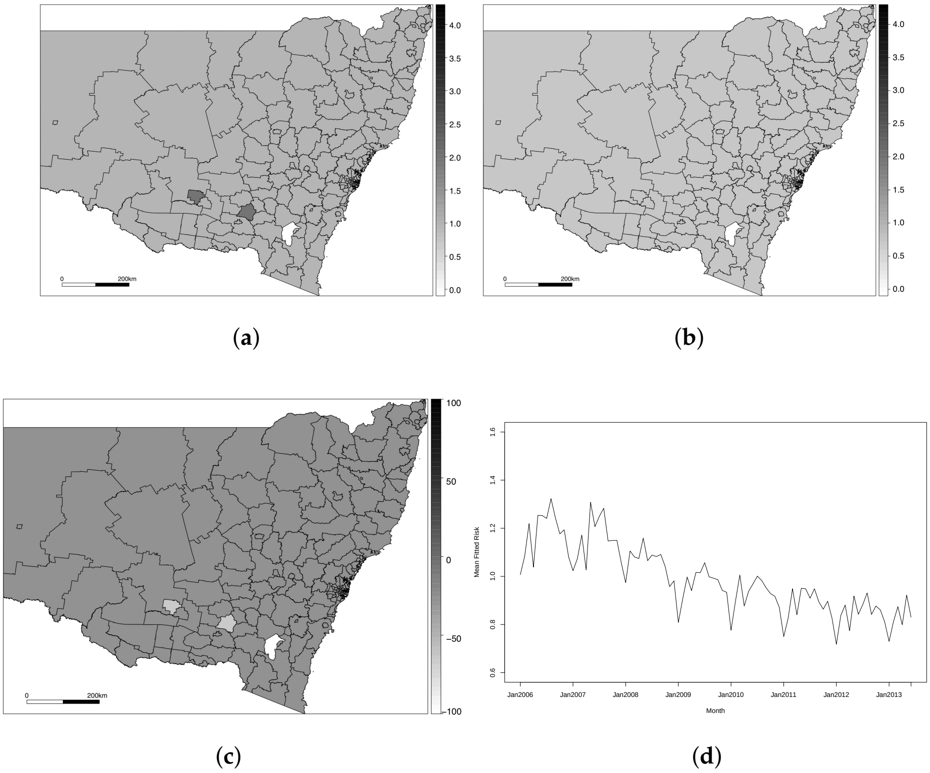

Figure S4. Results for Model 4. (**a**) Fitted IHD risks for January 2006. (**b**) Fitted IHD risks for January 2013. (**c**) Overall percentage change in fitted risks between January 2006 and January 2013. (**d**) Mean IHD risk in New South Wales by month.

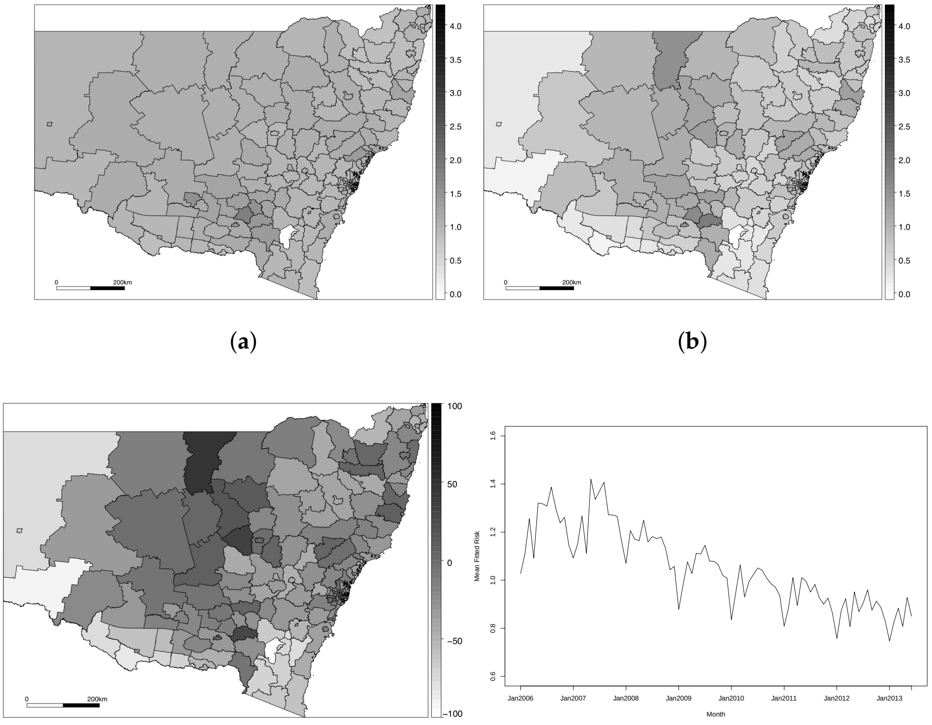

(**a**) (**b**) (**c**) (**d**)

Figure S5. Results for Model 5. (**a**) Fitted IHD risks for January 2006. (**b**) Fitted IHD risks for January 2013. (**c**) Overall percentage change in fitted risks between January 2006 and January 2013. (**d**) Mean IHD risk in New South Wales by month.

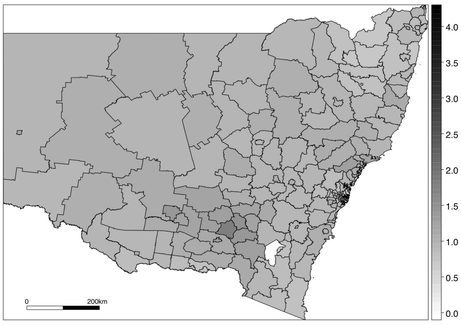

(**a**)

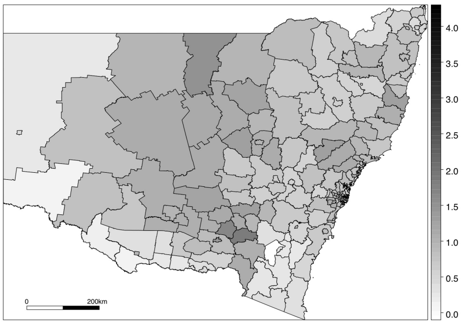

(**b**)

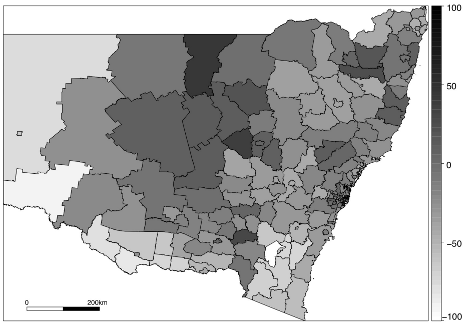

(**c**)

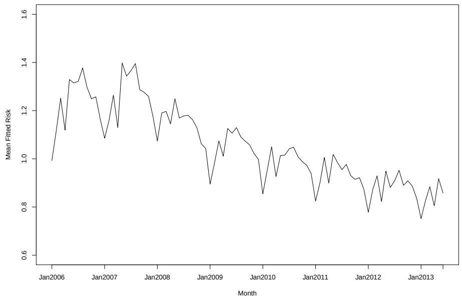

(**d**)

Figure S6. Results for Model 6. (**a**) Fitted IHD risks for January 2006. (**b**) Fitted IHD risks for January 2013. (**c**) Overall percentage change in fitted risks between January 2006 and January 2013. (**d**) Mean IHD risk in New South Wales by month.

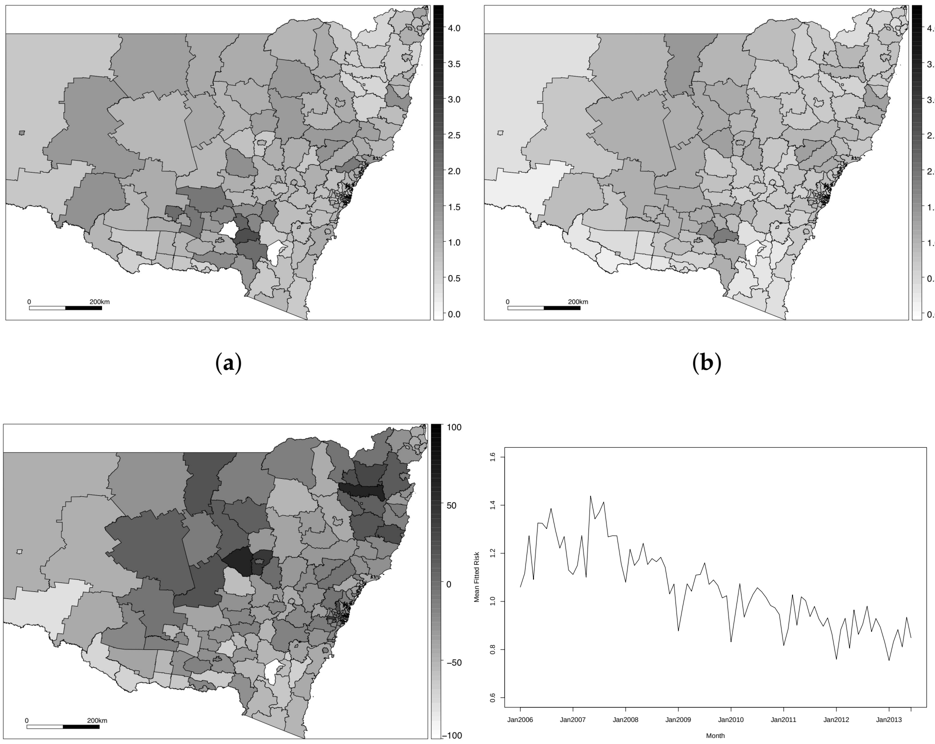

(**a**) (**b**)

(**c**) (**d**)

Figure S7. Results for Model 7. (**a**) Fitted IHD risks for January 2006. (**b**) Fitted IHD risks for January 2013. (**c**) Overall percentage change in fitted risks between January 2006 and January 2013. (**d**) Mean IHD risk in New South Wales by month.
