# Supplementary figures and images for: A Comparison of Spatio-Temporal Disease Mapping Approaches Including an Application to Ischaemic Heart Disease in New South Wales, Australia
Source: Int J Environ Res Public Health. 2017 Feb 3;14(2):146. doi: 10.3390/ijerph14020146 (PMC5334700; doi:10.3390/ijerph14020146)

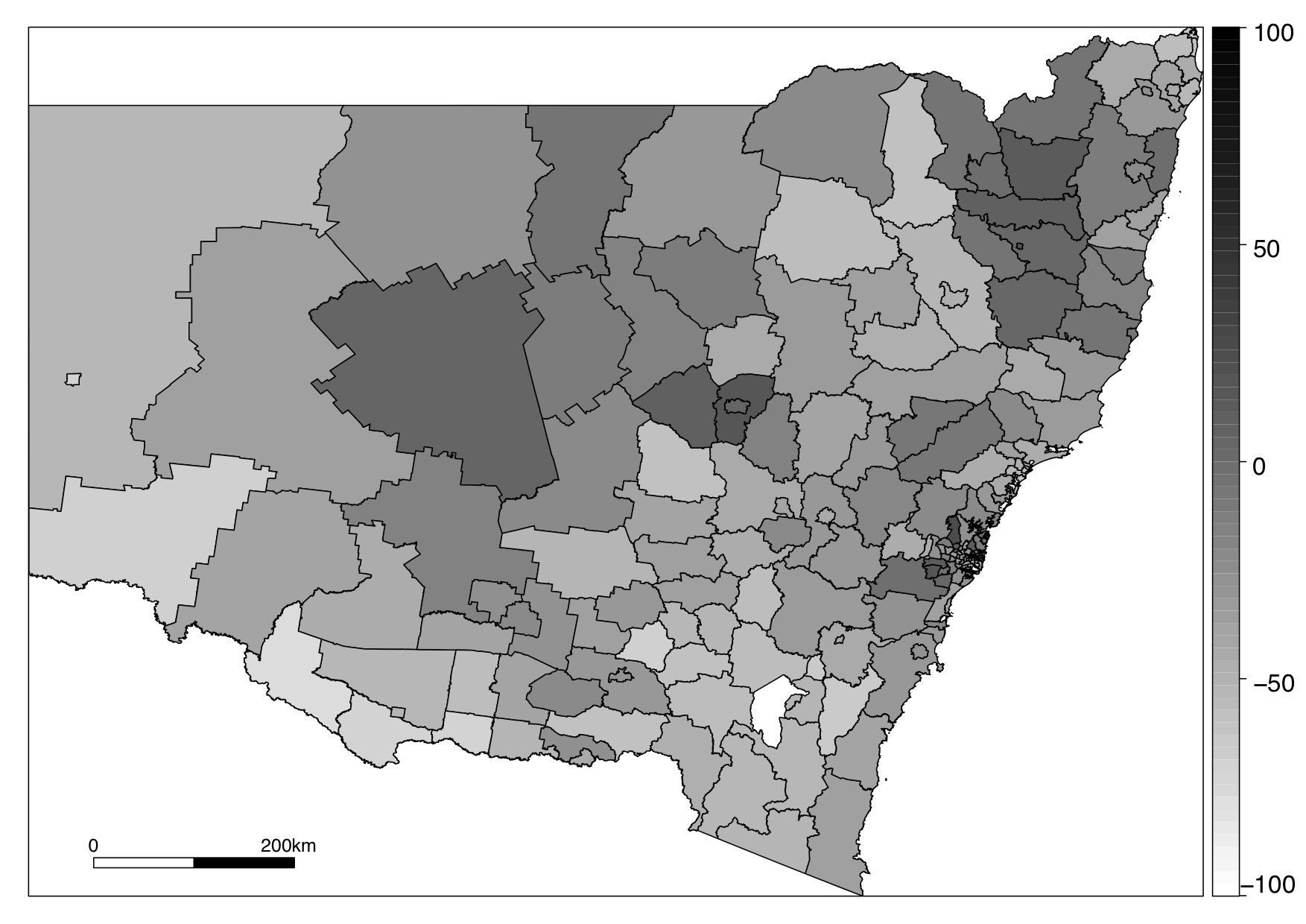

Supplement: Supplementary file 1 [file ijerph-14-00146-s001.zip › Supplementary/Figures/Mod1Change.jpg]

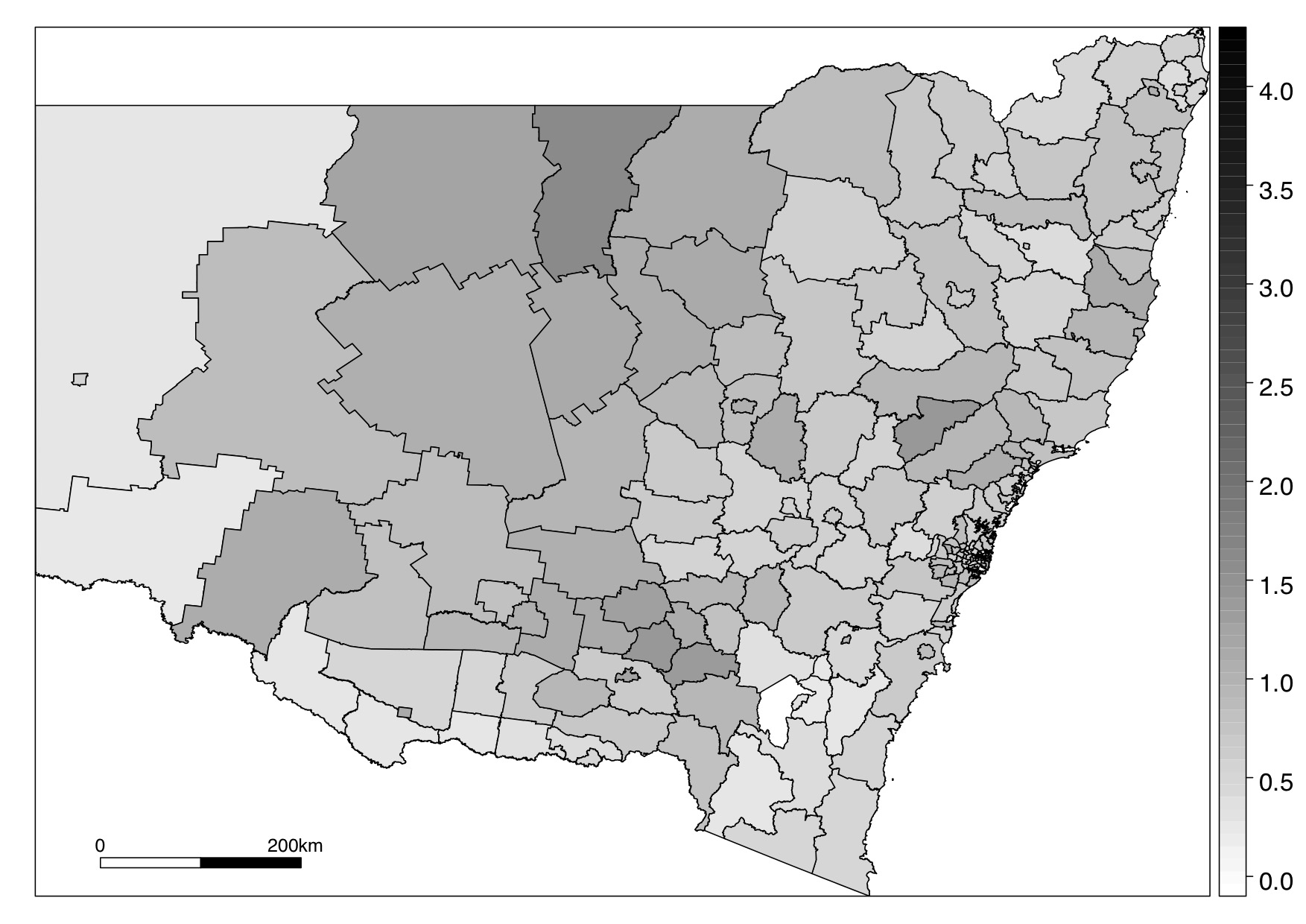

Supplement: Supplementary file 1 [file ijerph-14-00146-s001.zip › Supplementary/Figures/Mod1Final.jpg]

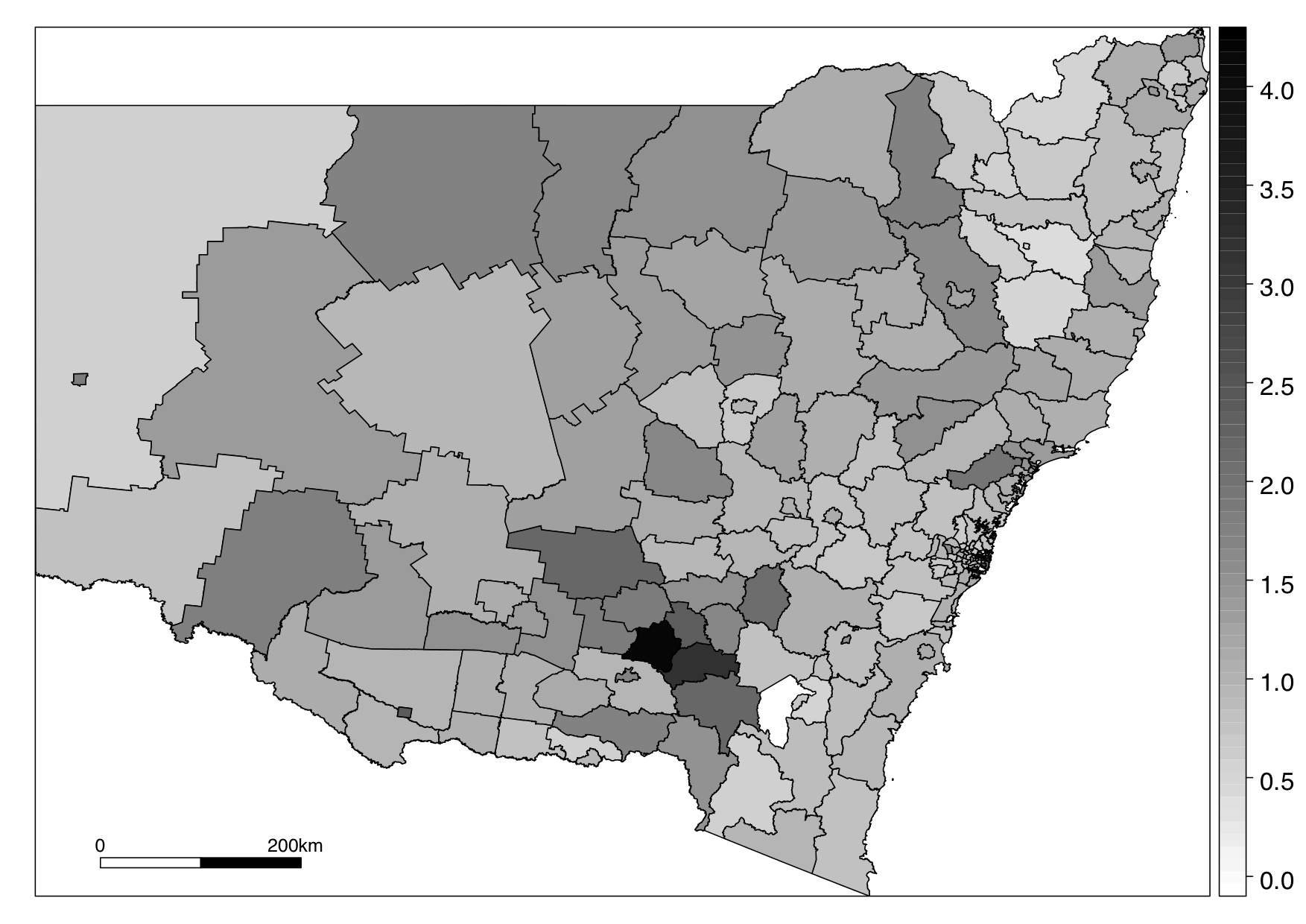

Supplement: Supplementary file 1 [file ijerph-14-00146-s001.zip › Supplementary/Figures/Mod1Start.jpg]

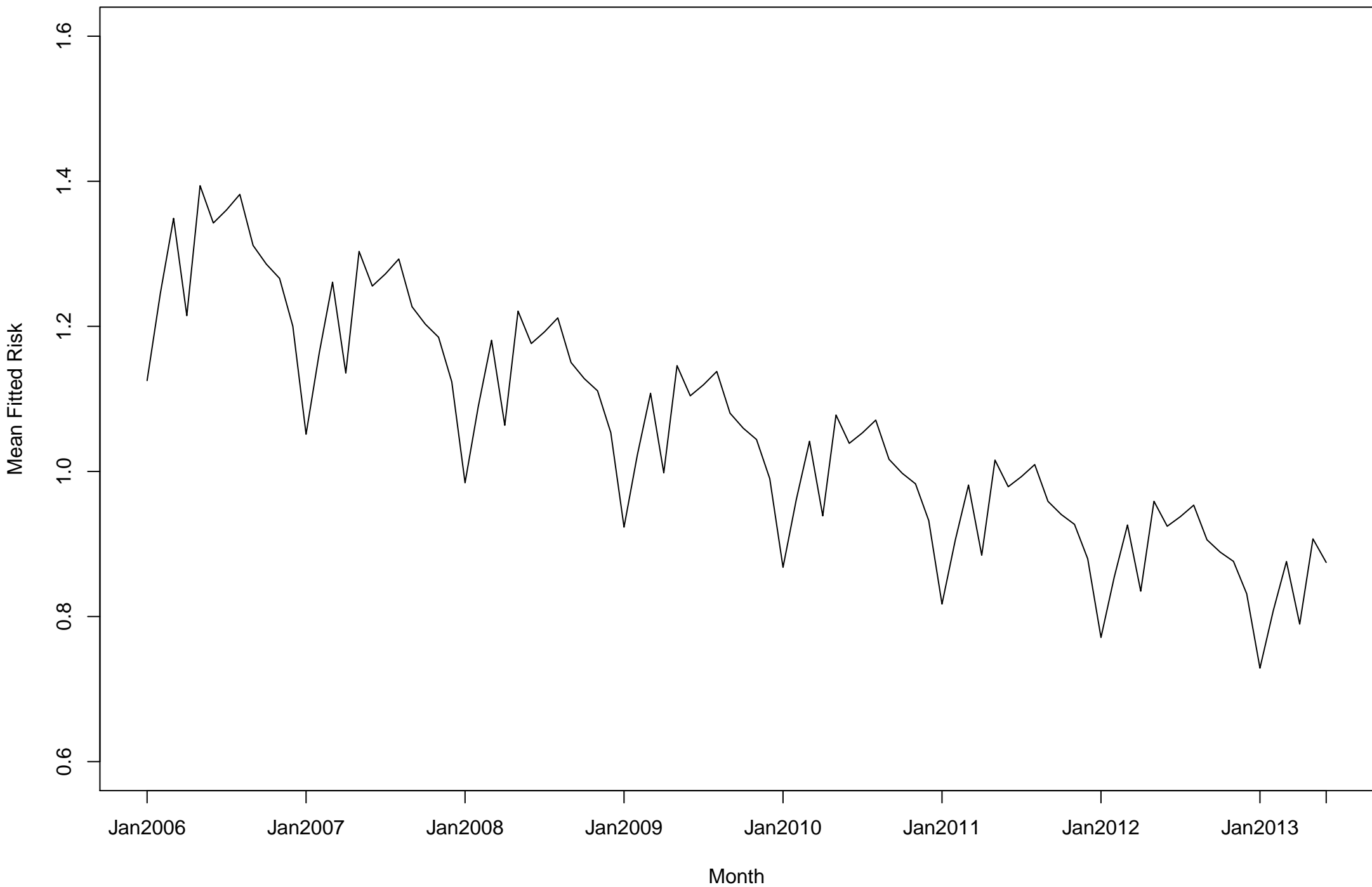

Supplement: Supplementary file 1 [file ijerph-14-00146-s001.zip › Supplementary/Figures/Mod1Trend.pdf]

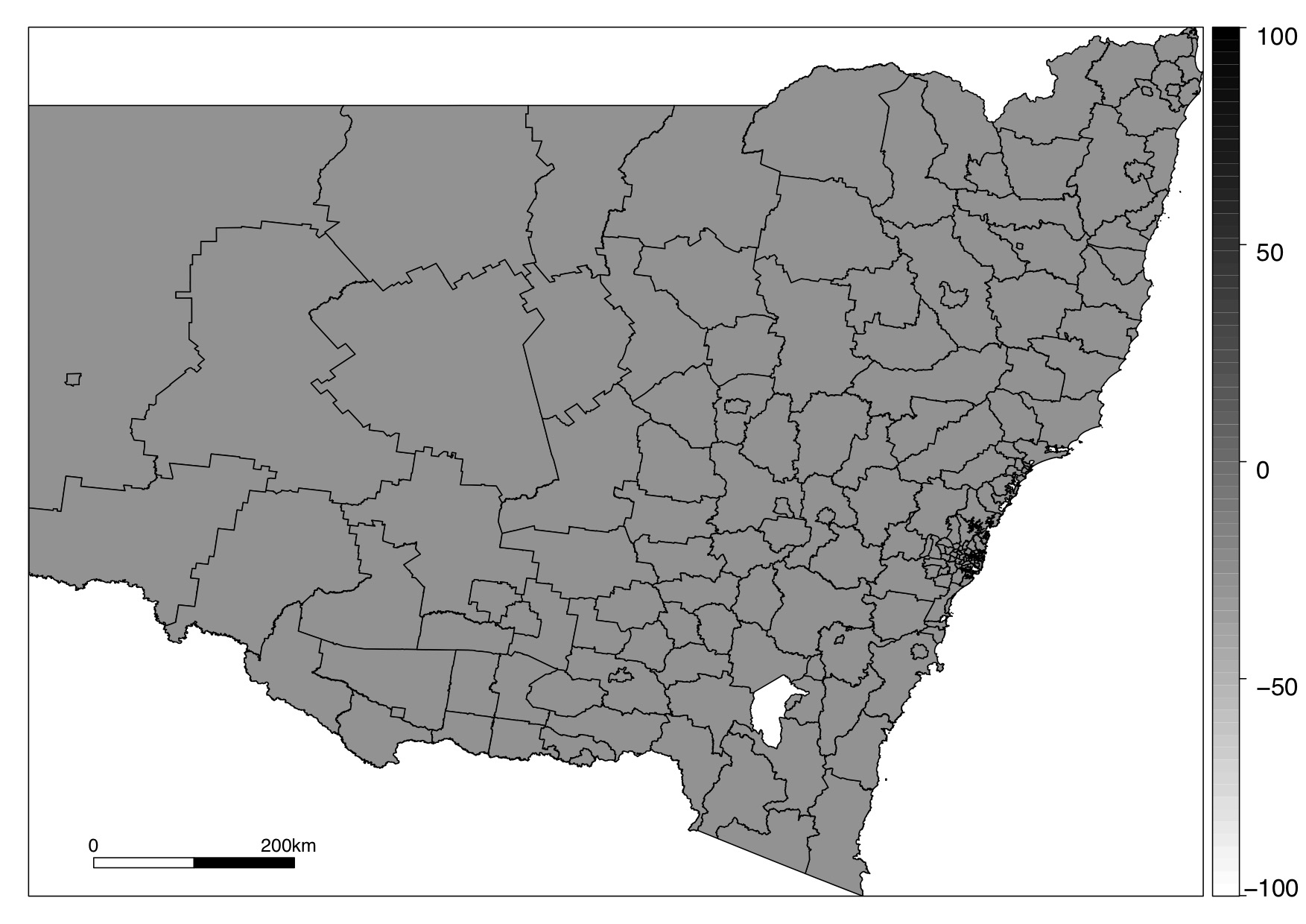

Supplement: Supplementary file 1 [file ijerph-14-00146-s001.zip › Supplementary/Figures/Mod2Change.jpg]

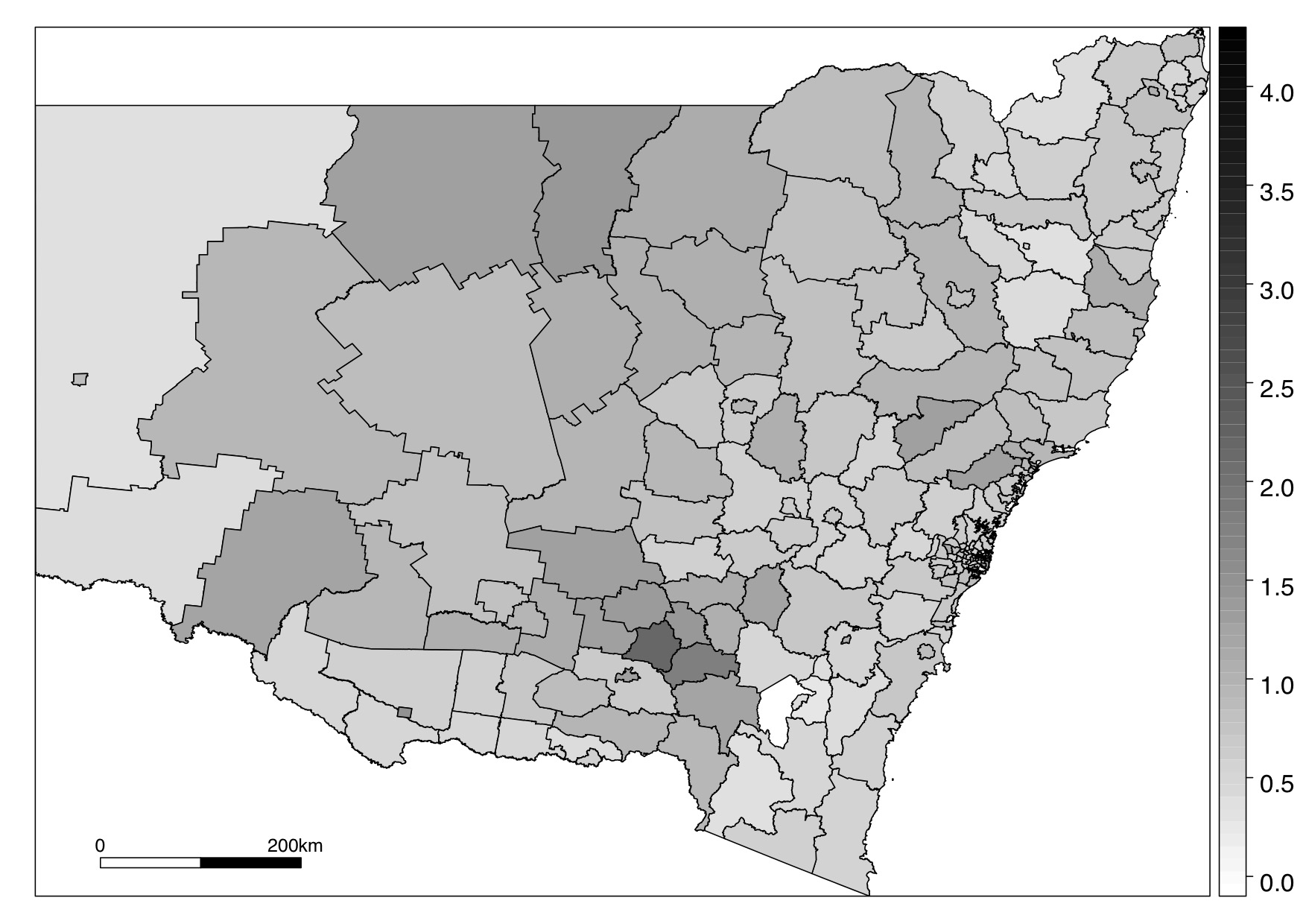

Supplement: Supplementary file 1 [file ijerph-14-00146-s001.zip › Supplementary/Figures/Mod2Final.jpg]

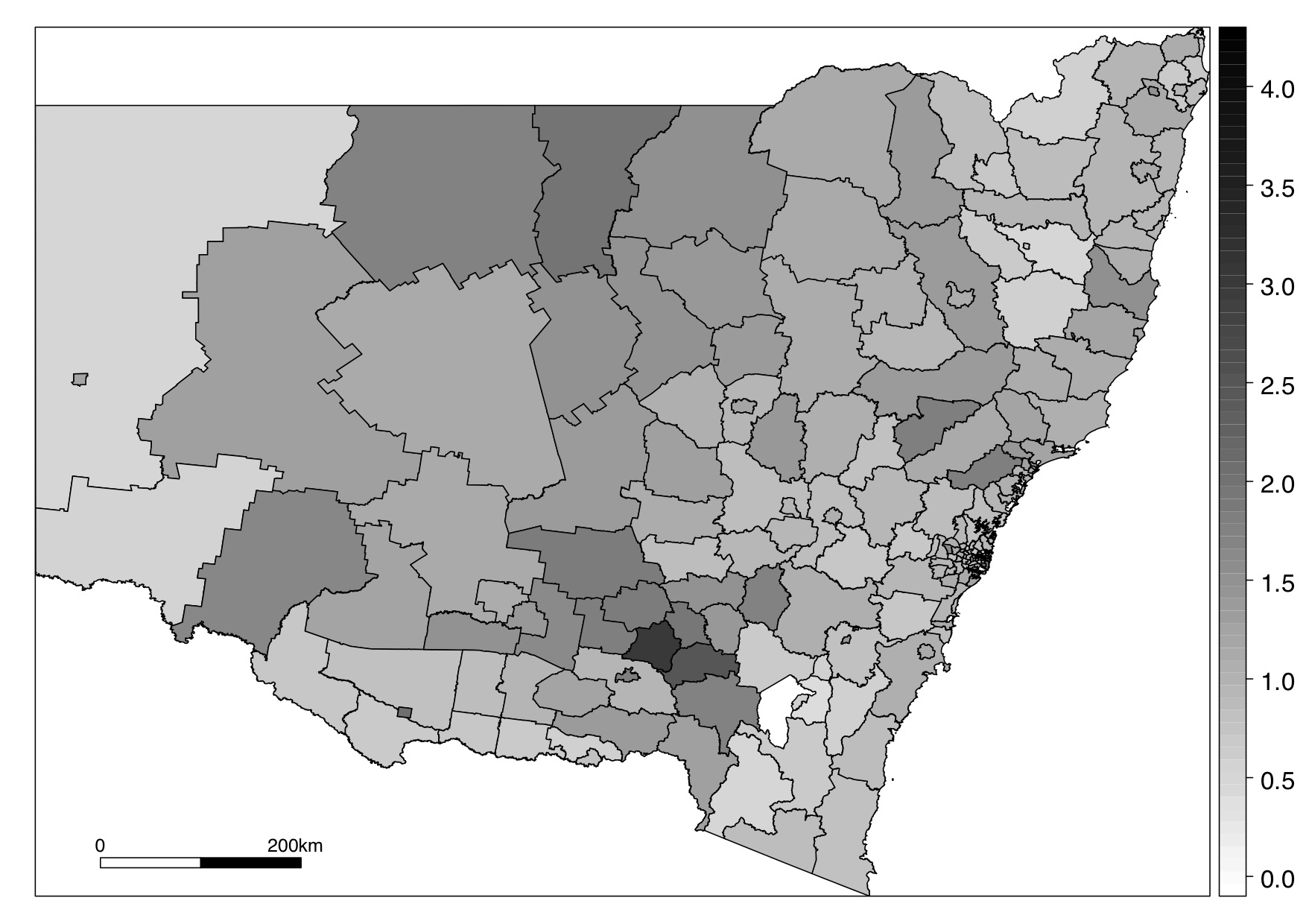

Supplement: Supplementary file 1 [file ijerph-14-00146-s001.zip › Supplementary/Figures/Mod2Start.jpg]

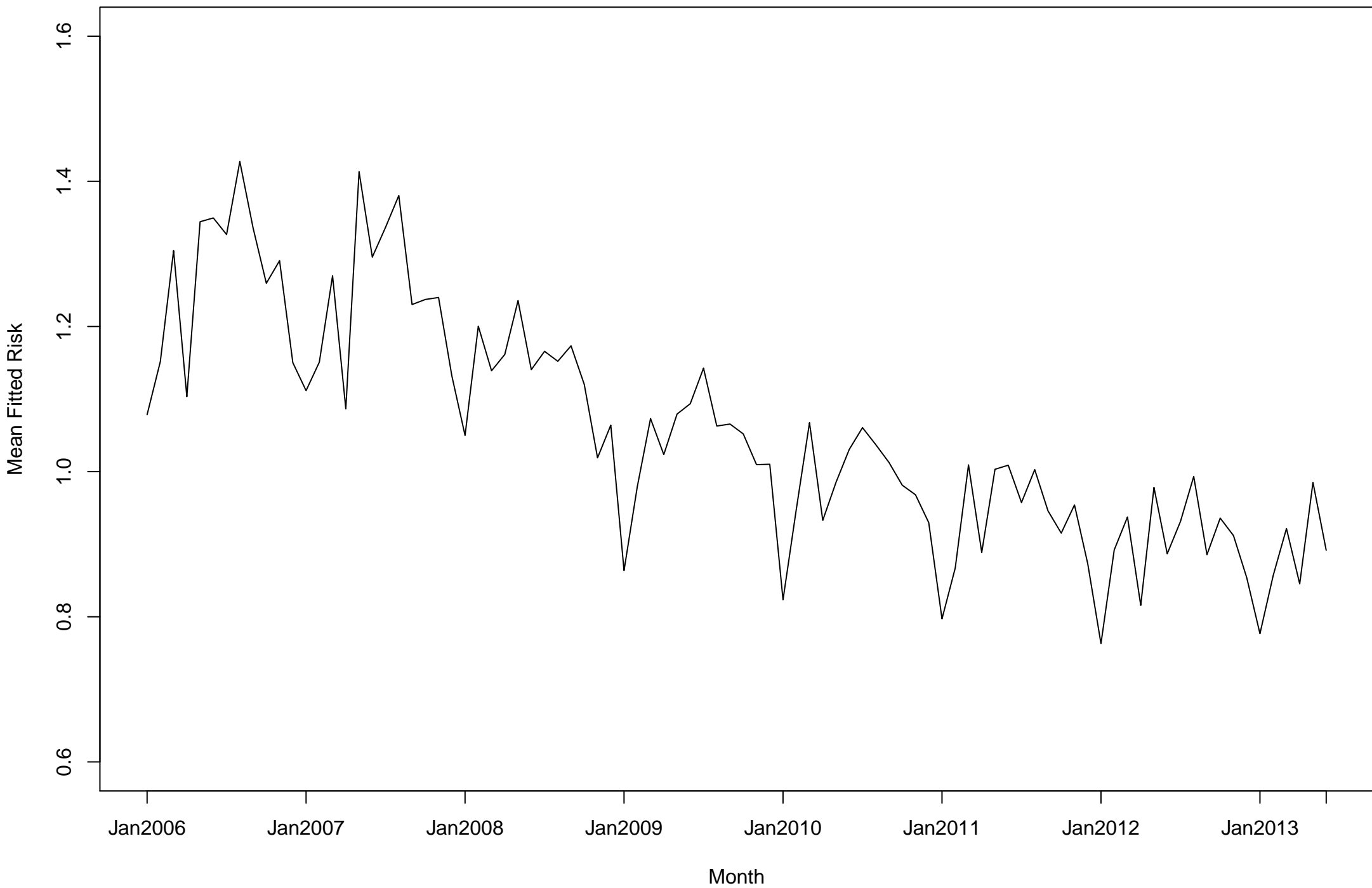

Supplement: Supplementary file 1 [file ijerph-14-00146-s001.zip › Supplementary/Figures/Mod2Trend.pdf]

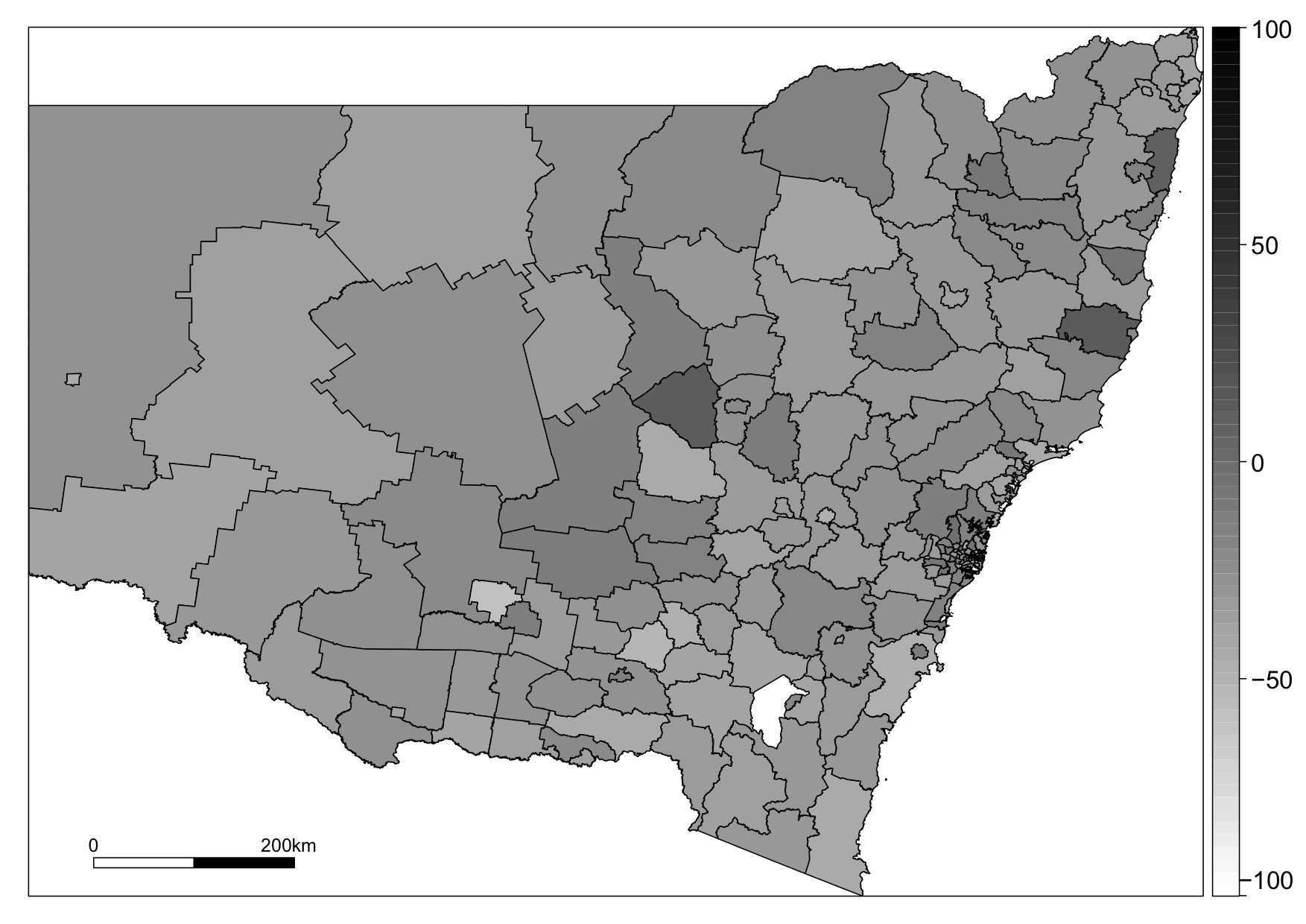

Supplement: Supplementary file 1 [file ijerph-14-00146-s001.zip › Supplementary/Figures/Mod3Change.jpg]

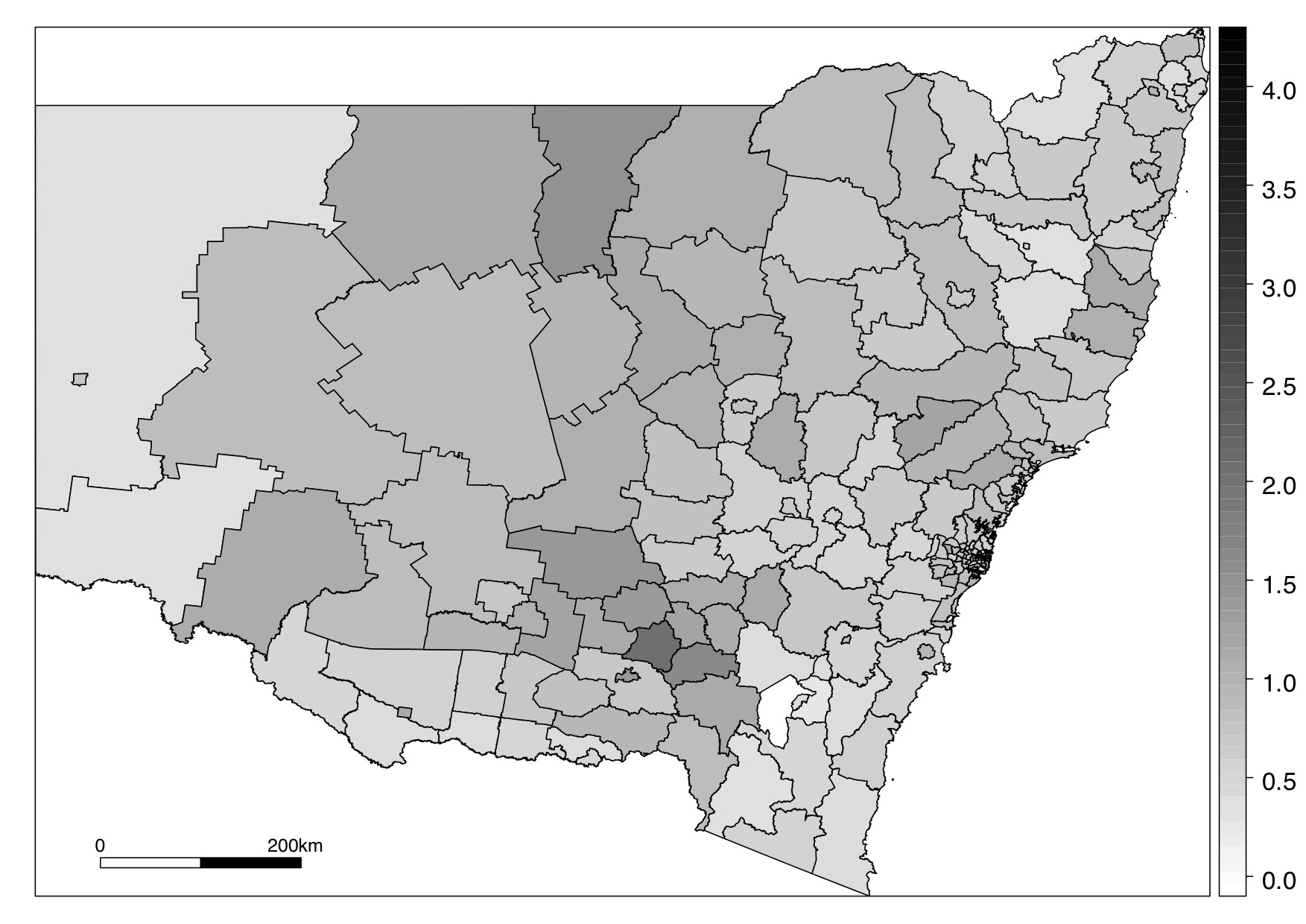

Supplement: Supplementary file 1 [file ijerph-14-00146-s001.zip › Supplementary/Figures/Mod3Final.jpg]

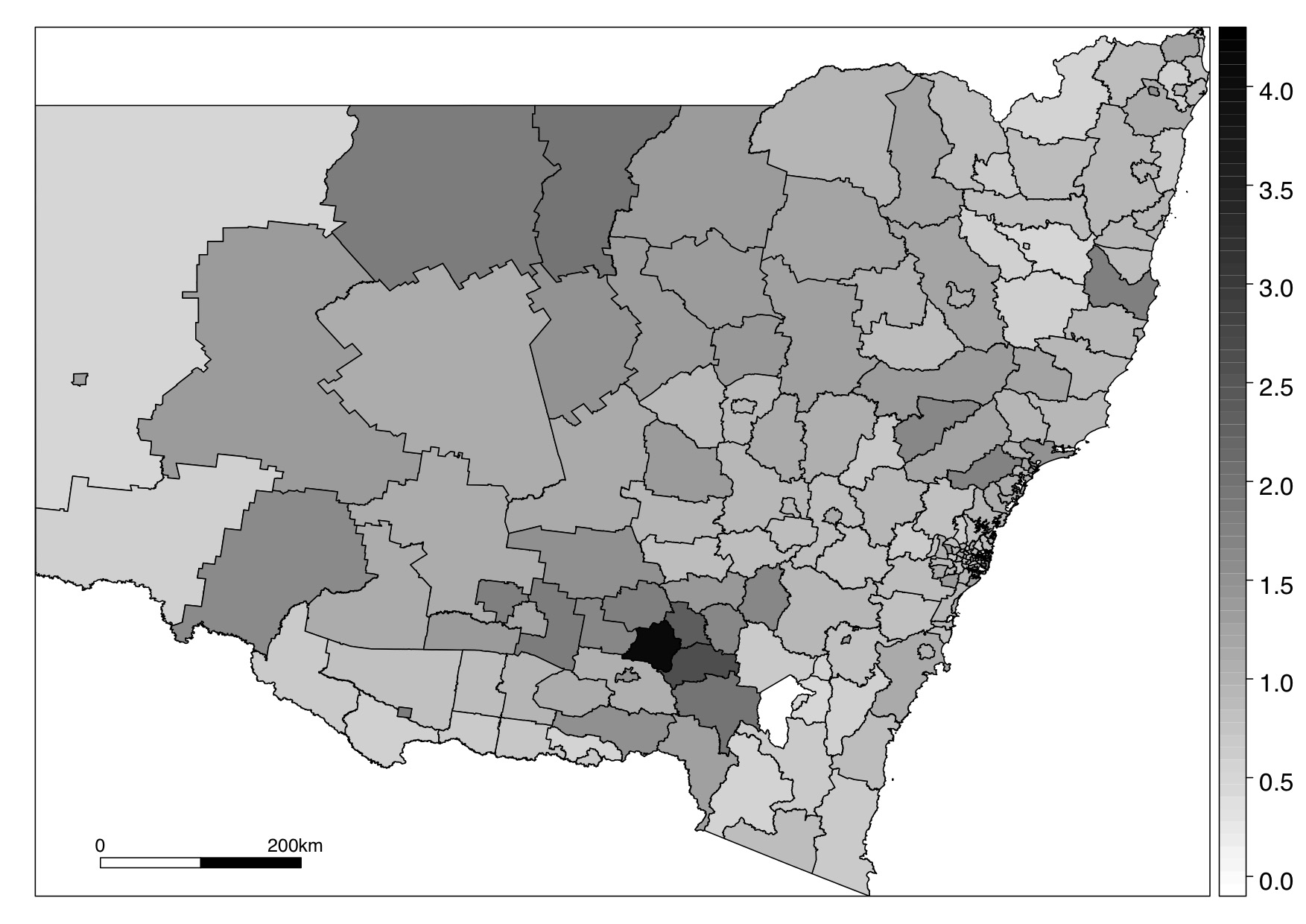

Supplement: Supplementary file 1 [file ijerph-14-00146-s001.zip › Supplementary/Figures/Mod3Start.jpg]

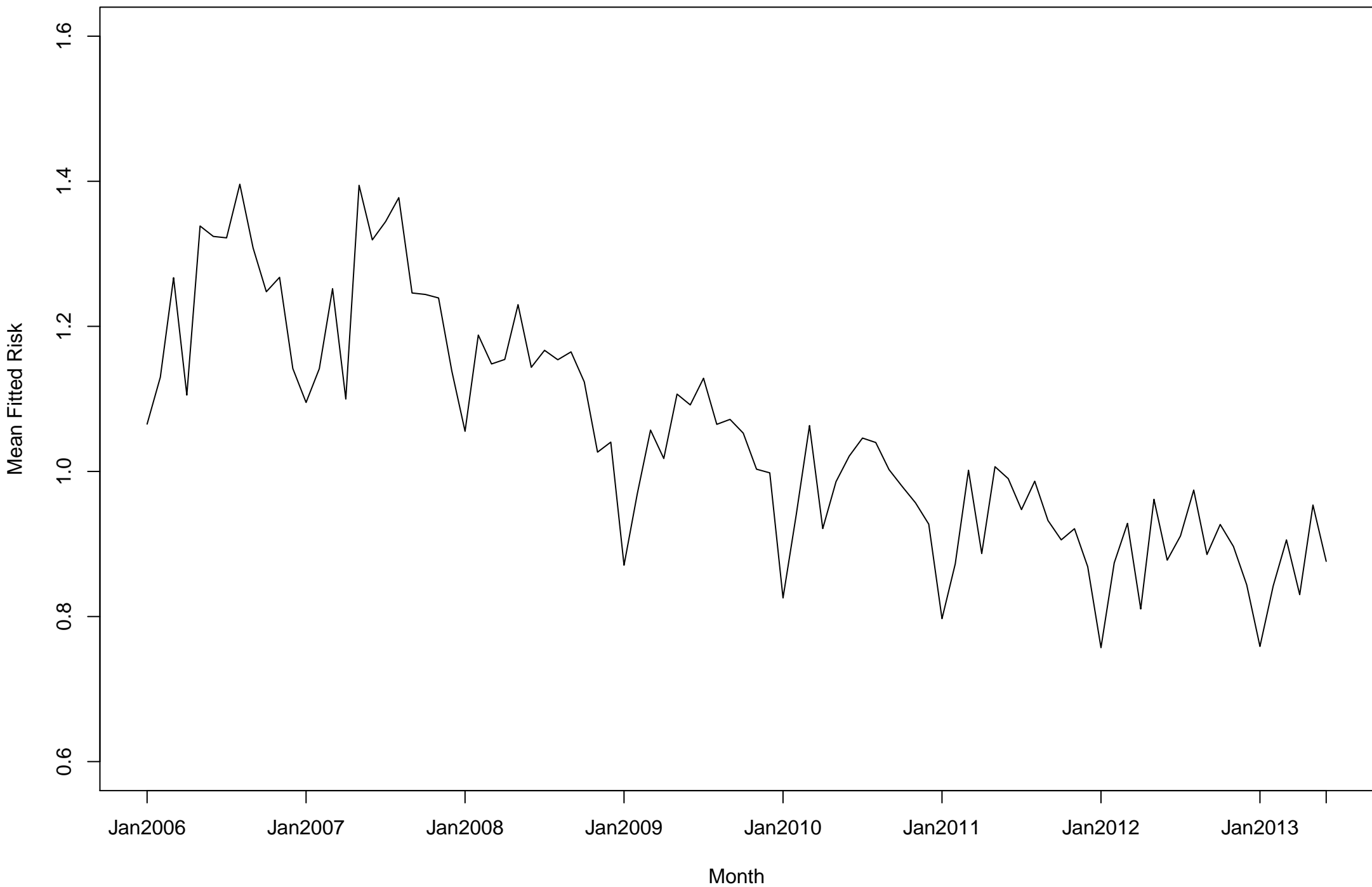

Supplement: Supplementary file 1 [file ijerph-14-00146-s001.zip › Supplementary/Figures/Mod3Trend.pdf]

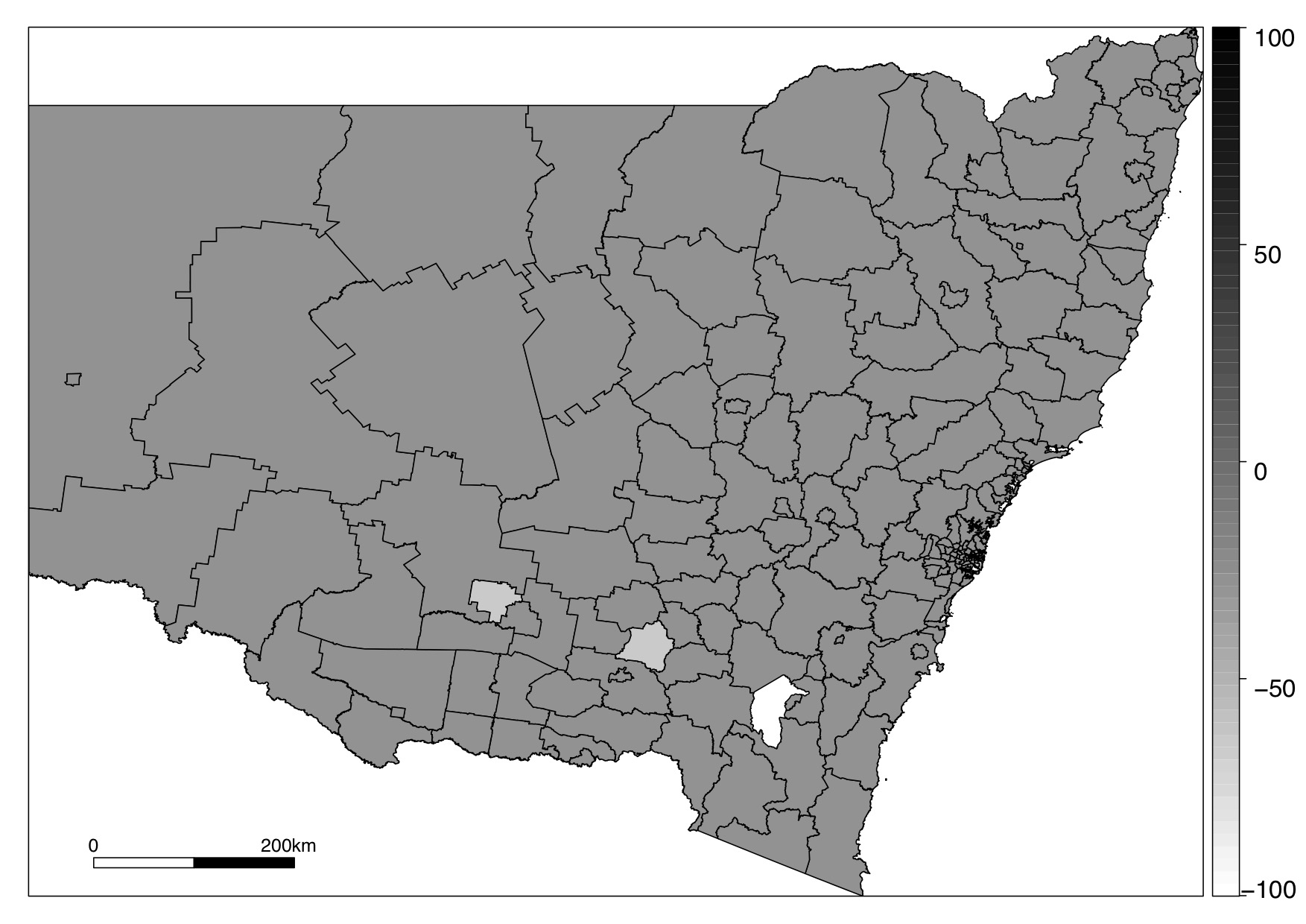

Supplement: Supplementary file 1 [file ijerph-14-00146-s001.zip › Supplementary/Figures/Mod4Change.jpg]

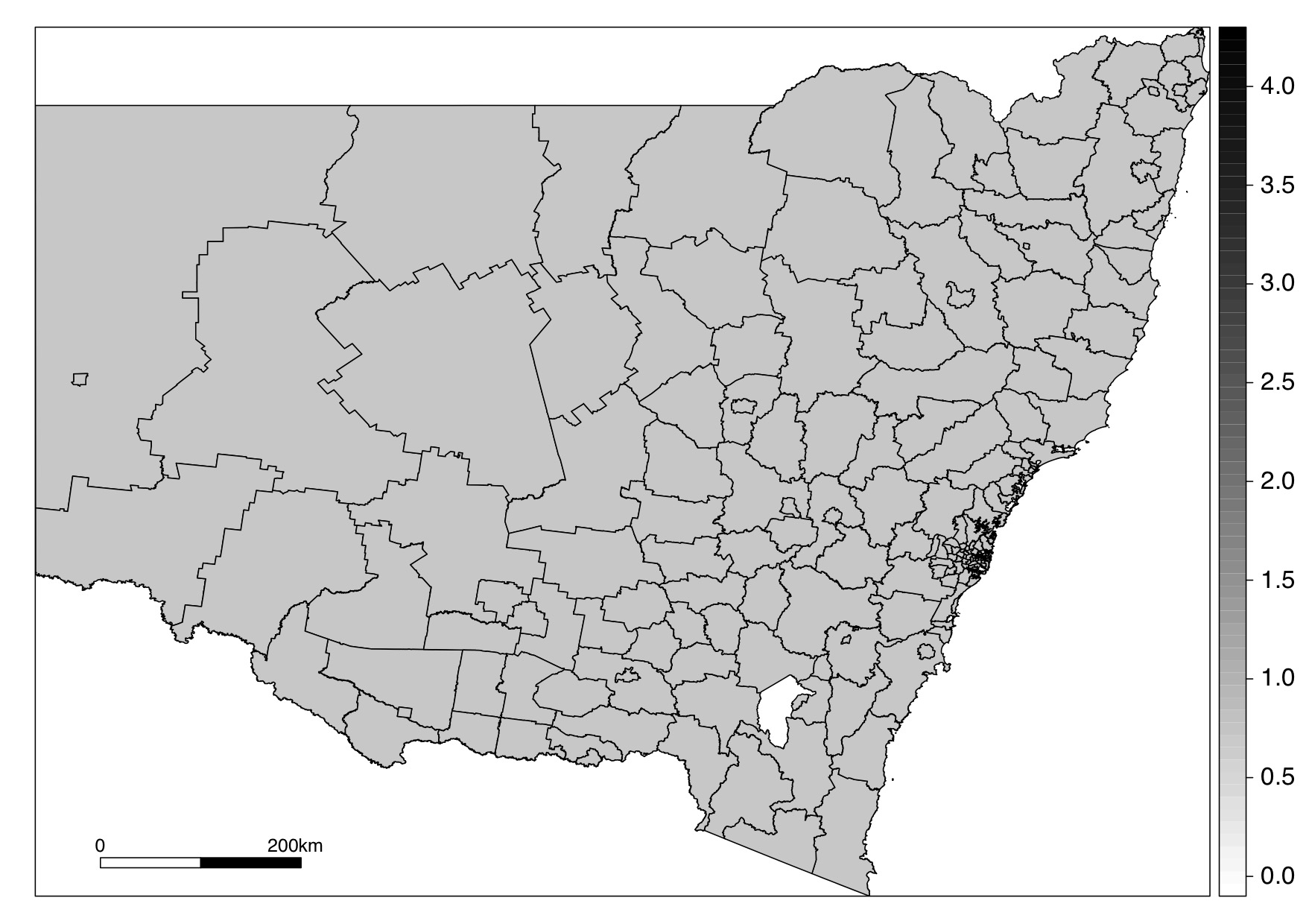

Supplement: Supplementary file 1 [file ijerph-14-00146-s001.zip › Supplementary/Figures/Mod4Final.jpg]

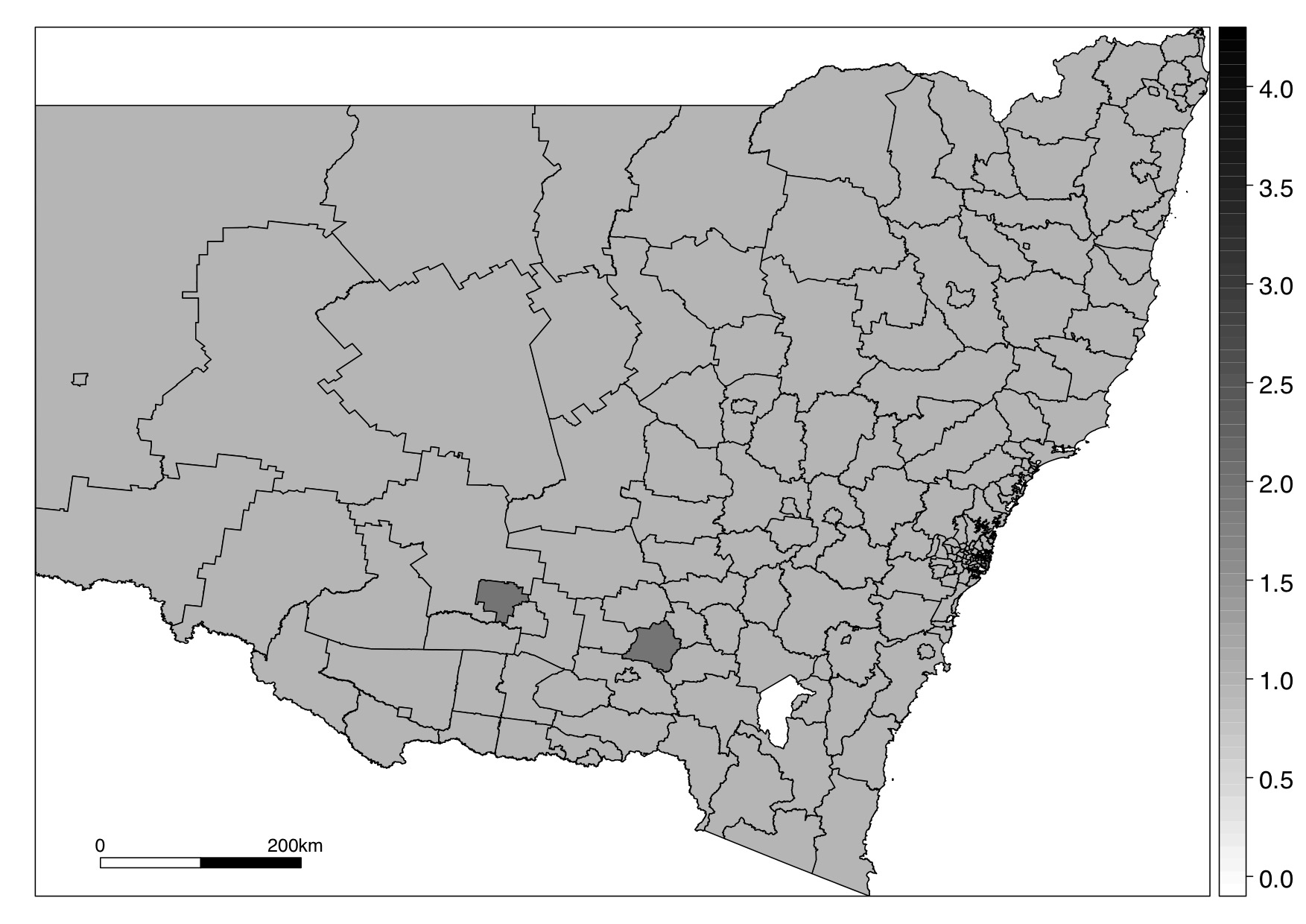

Supplement: Supplementary file 1 [file ijerph-14-00146-s001.zip › Supplementary/Figures/Mod4Start.jpg]

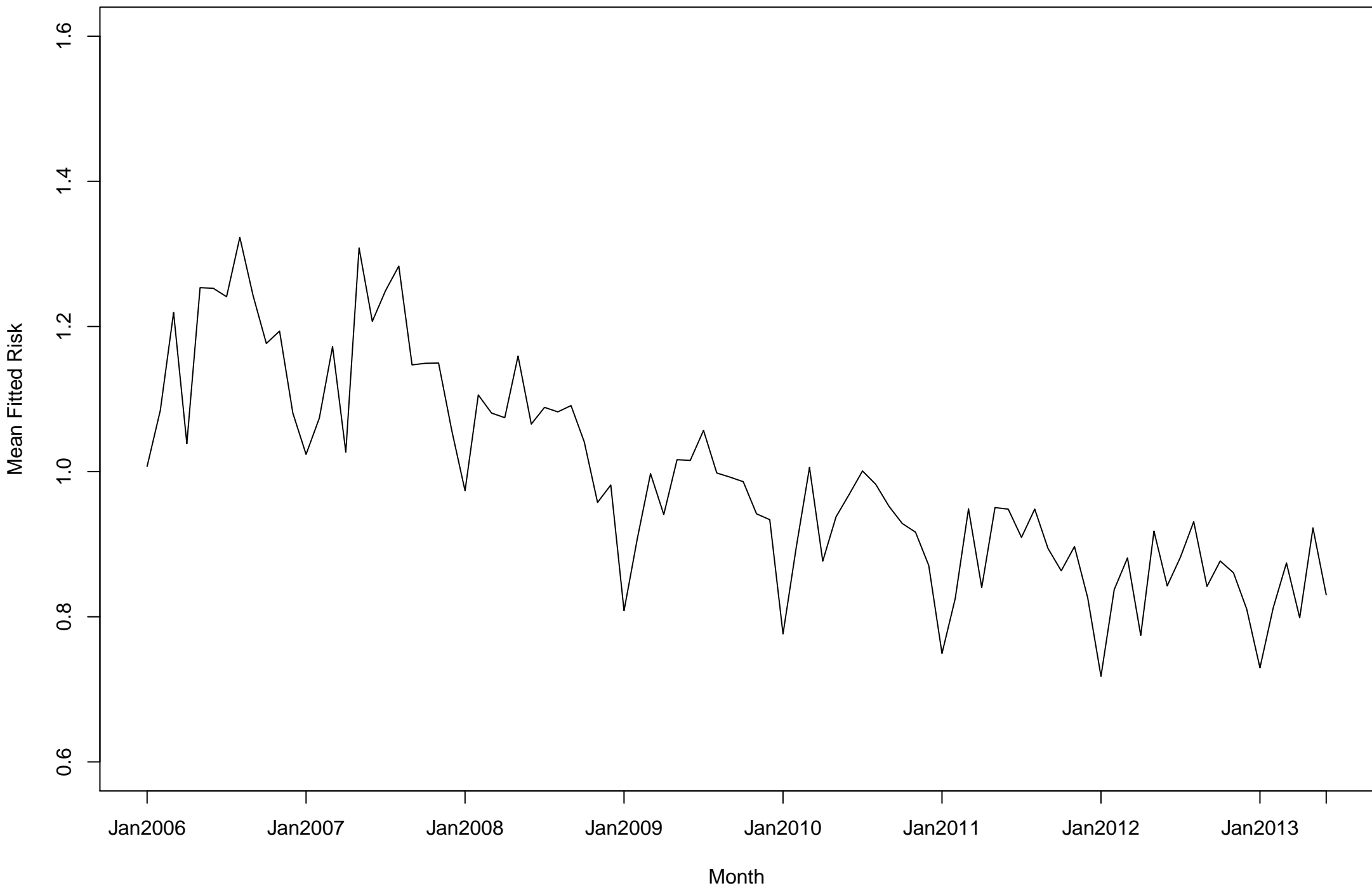

Supplement: Supplementary file 1 [file ijerph-14-00146-s001.zip › Supplementary/Figures/Mod4Trend.pdf]

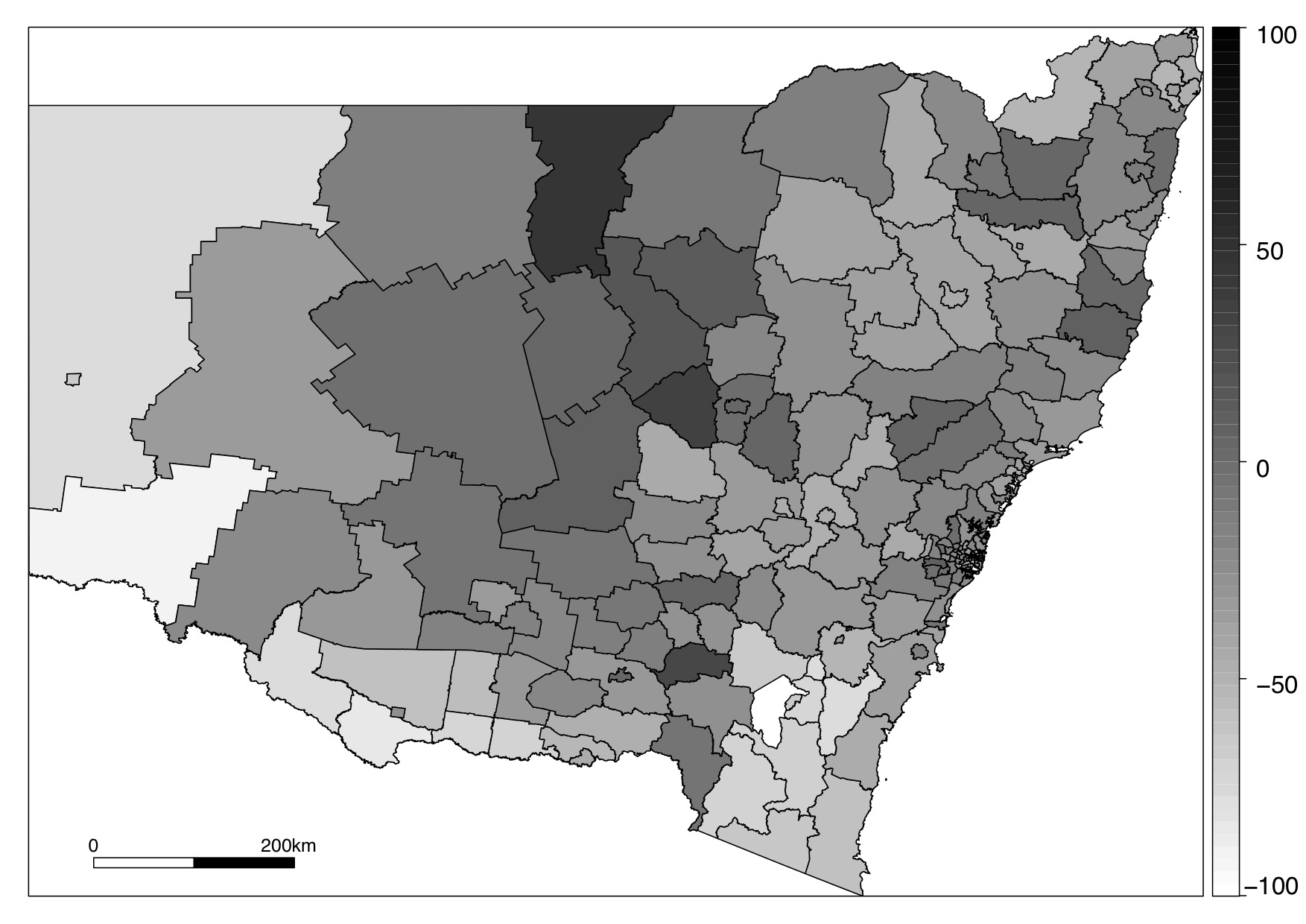

Supplement: Supplementary file 1 [file ijerph-14-00146-s001.zip › Supplementary/Figures/Mod5Change.jpg]

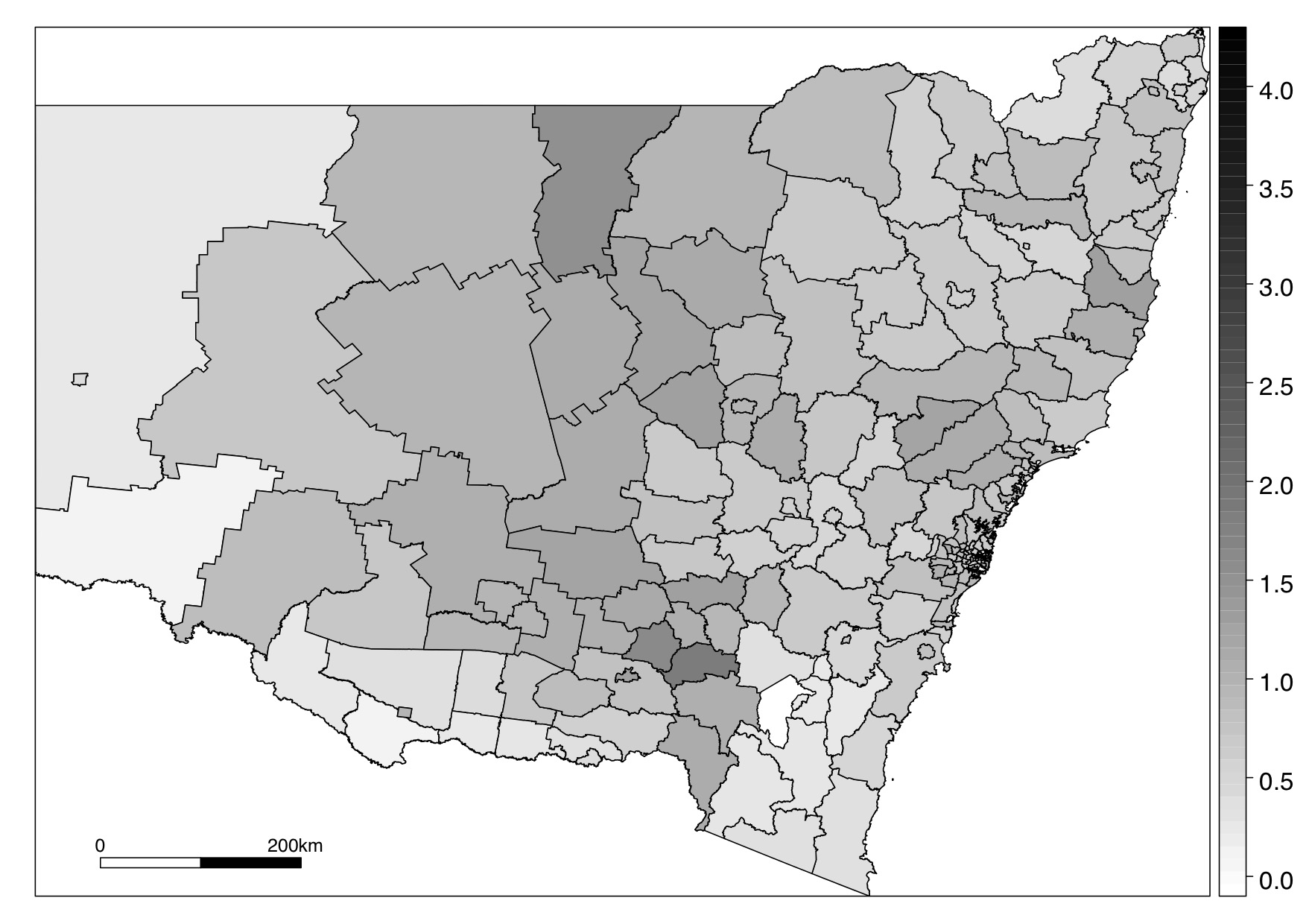

Supplement: Supplementary file 1 [file ijerph-14-00146-s001.zip › Supplementary/Figures/Mod5Final.jpg]

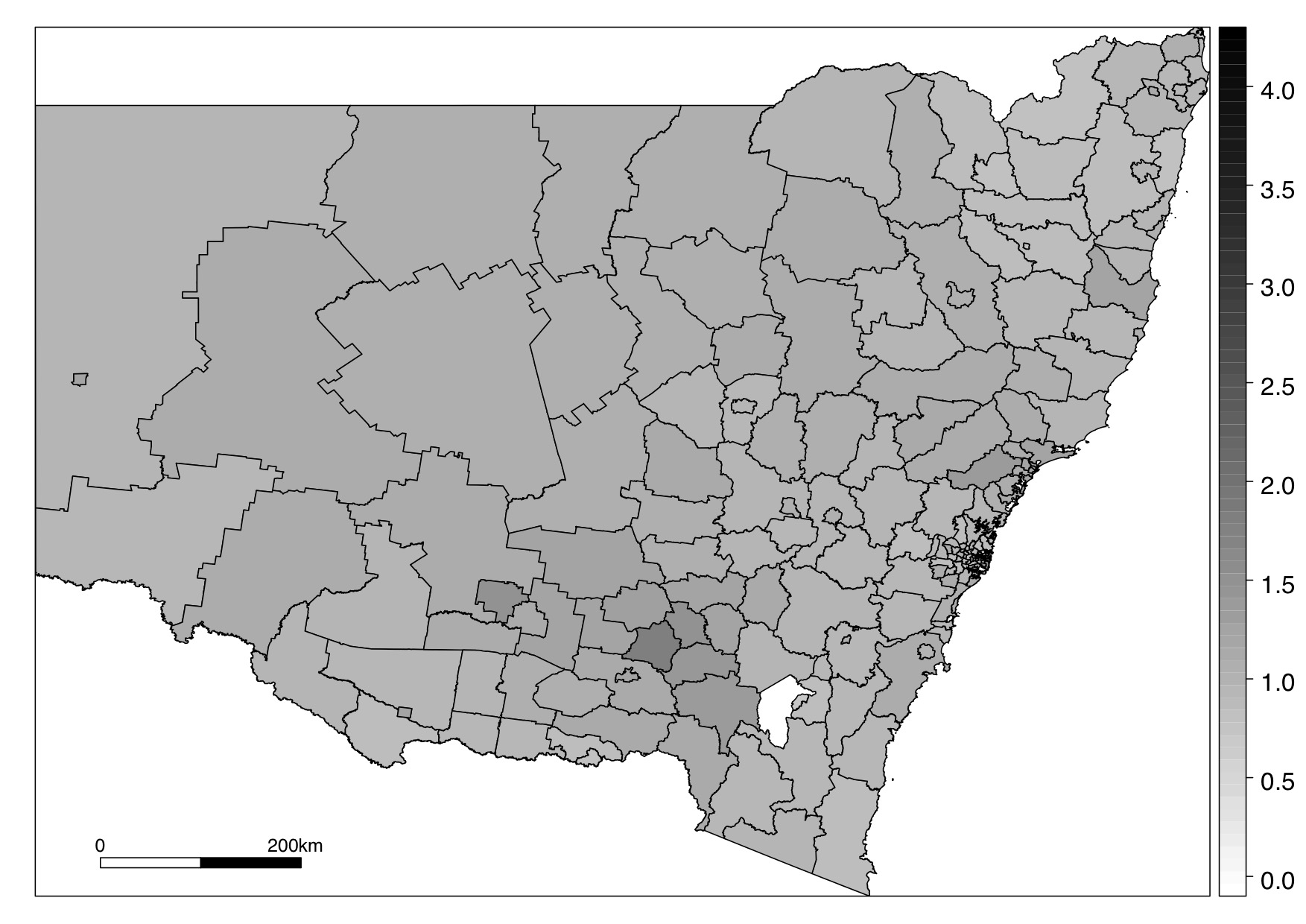

Supplement: Supplementary file 1 [file ijerph-14-00146-s001.zip › Supplementary/Figures/Mod5Start.jpg]

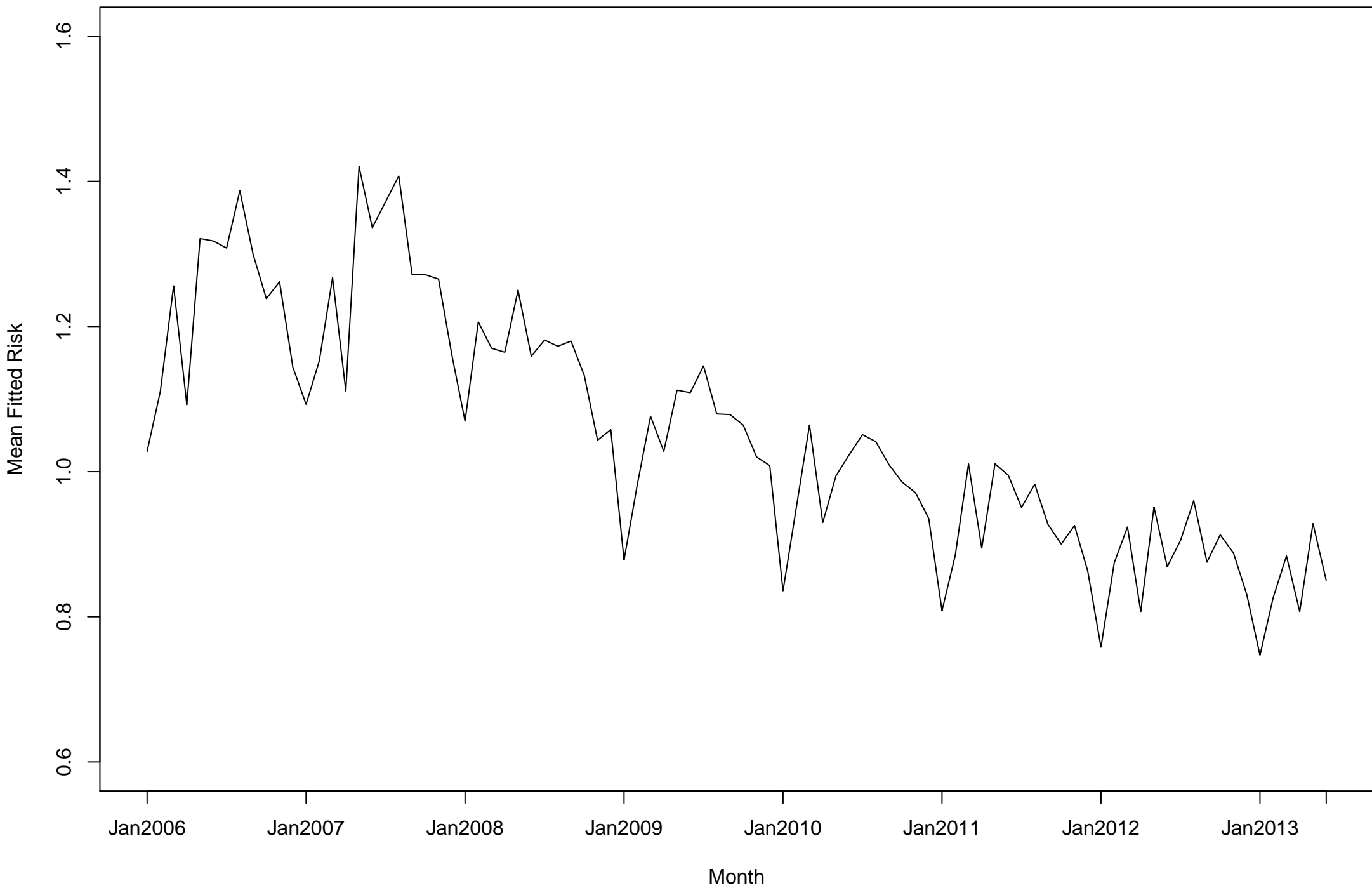

Supplement: Supplementary file 1 [file ijerph-14-00146-s001.zip › Supplementary/Figures/Mod5Trend.pdf]

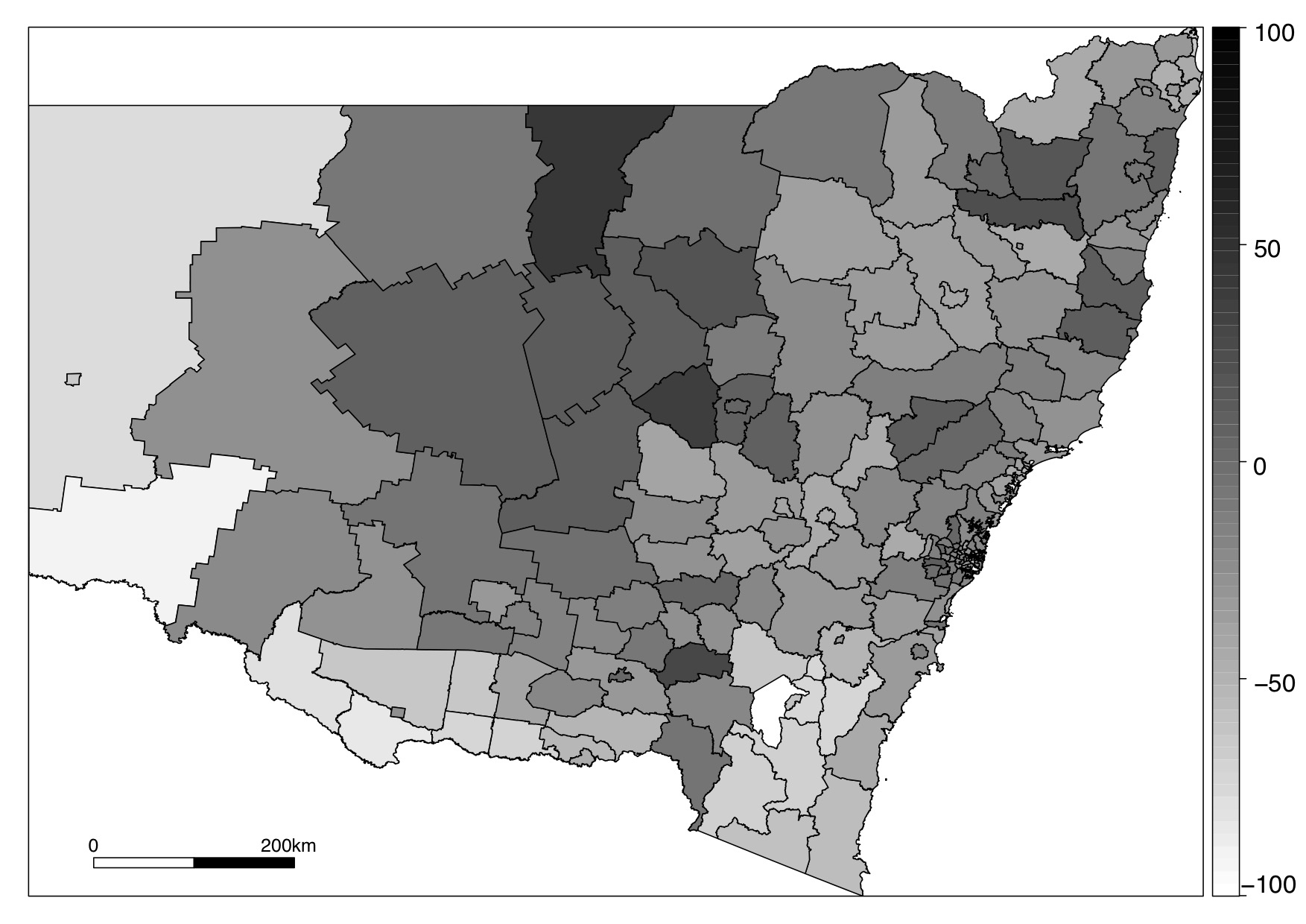

Supplement: Supplementary file 1 [file ijerph-14-00146-s001.zip › Supplementary/Figures/Mod6Change.jpg]

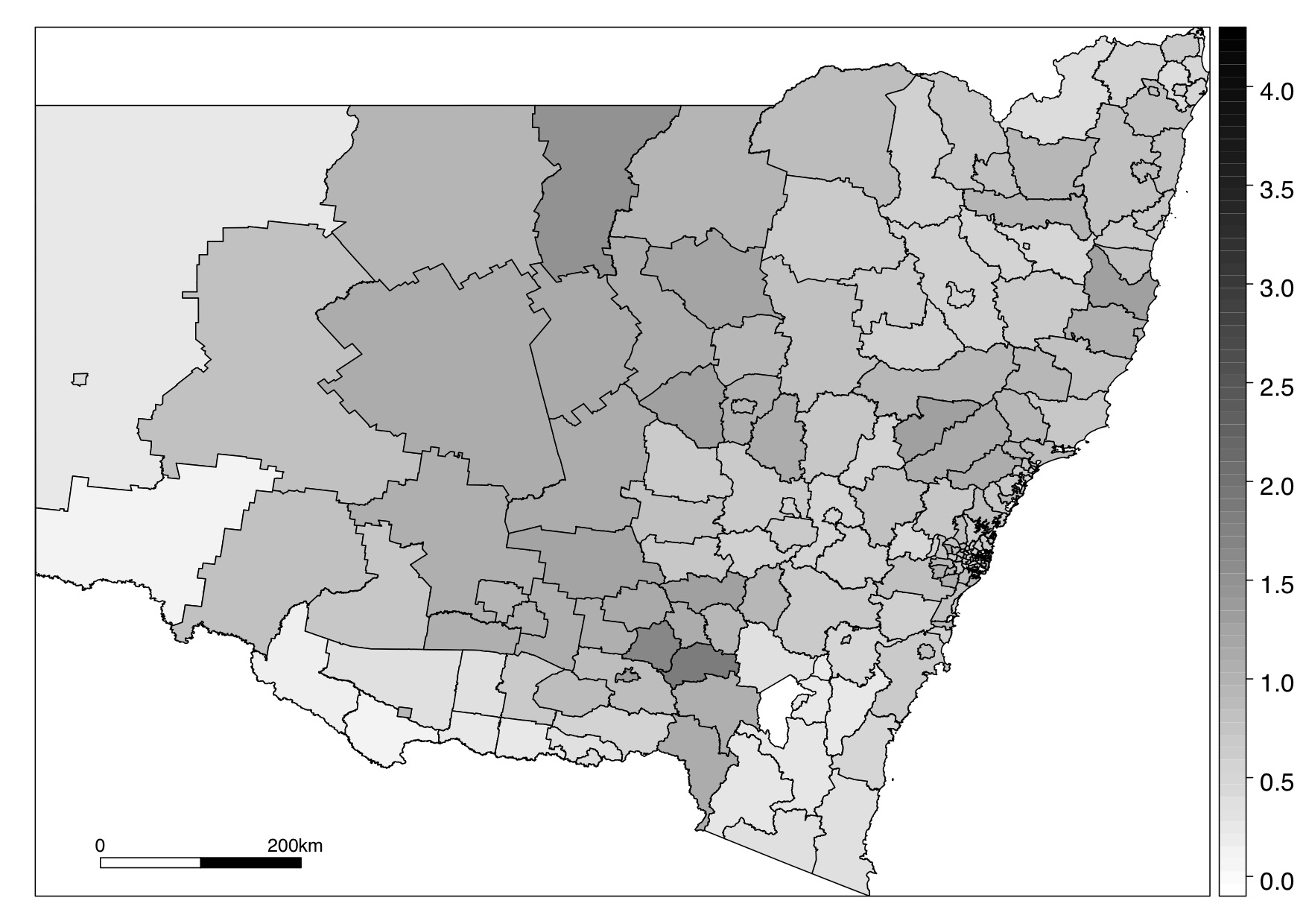

Supplement: Supplementary file 1 [file ijerph-14-00146-s001.zip › Supplementary/Figures/Mod6Final.jpg]

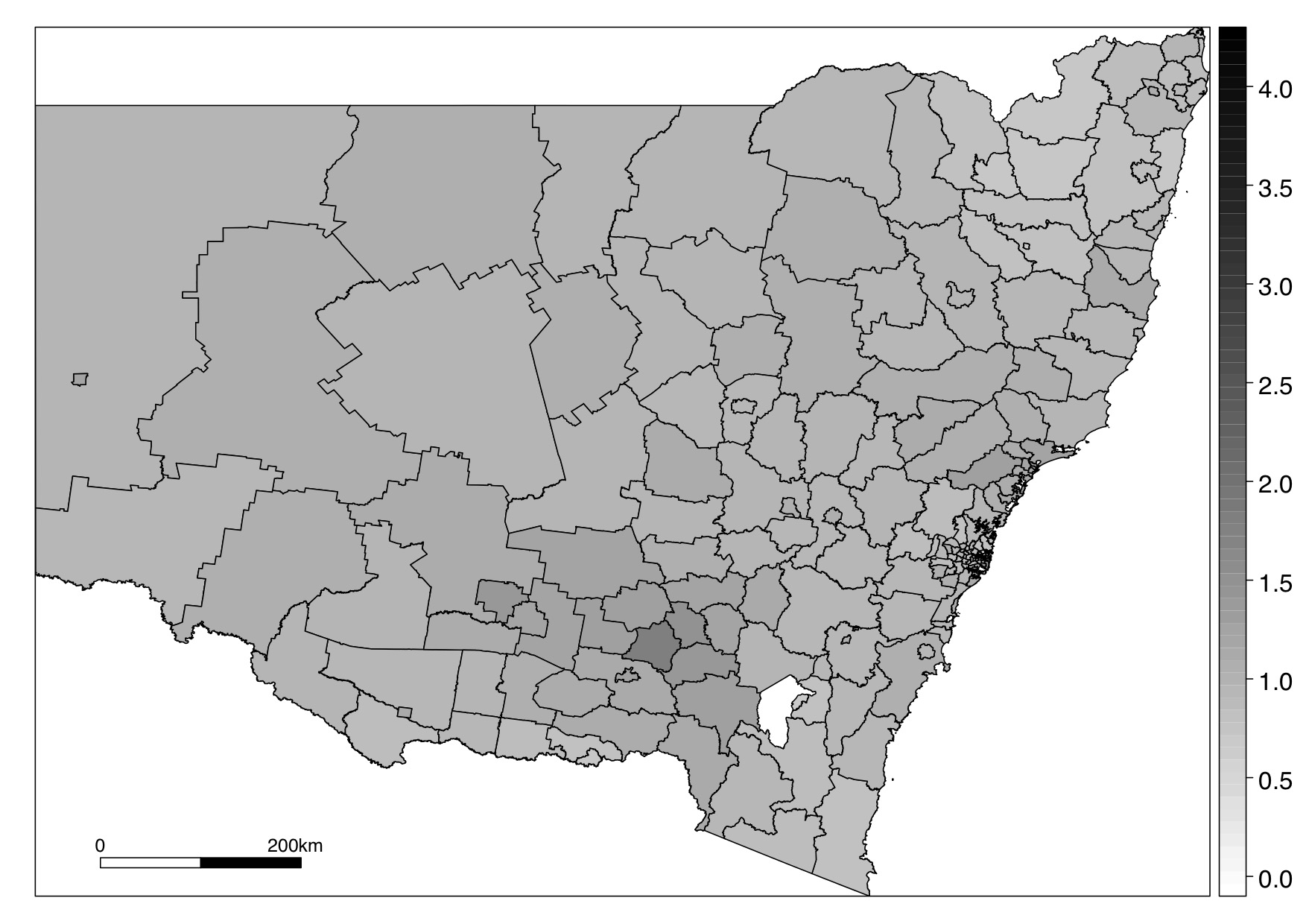

Supplement: Supplementary file 1 [file ijerph-14-00146-s001.zip › Supplementary/Figures/Mod6Start.jpg]

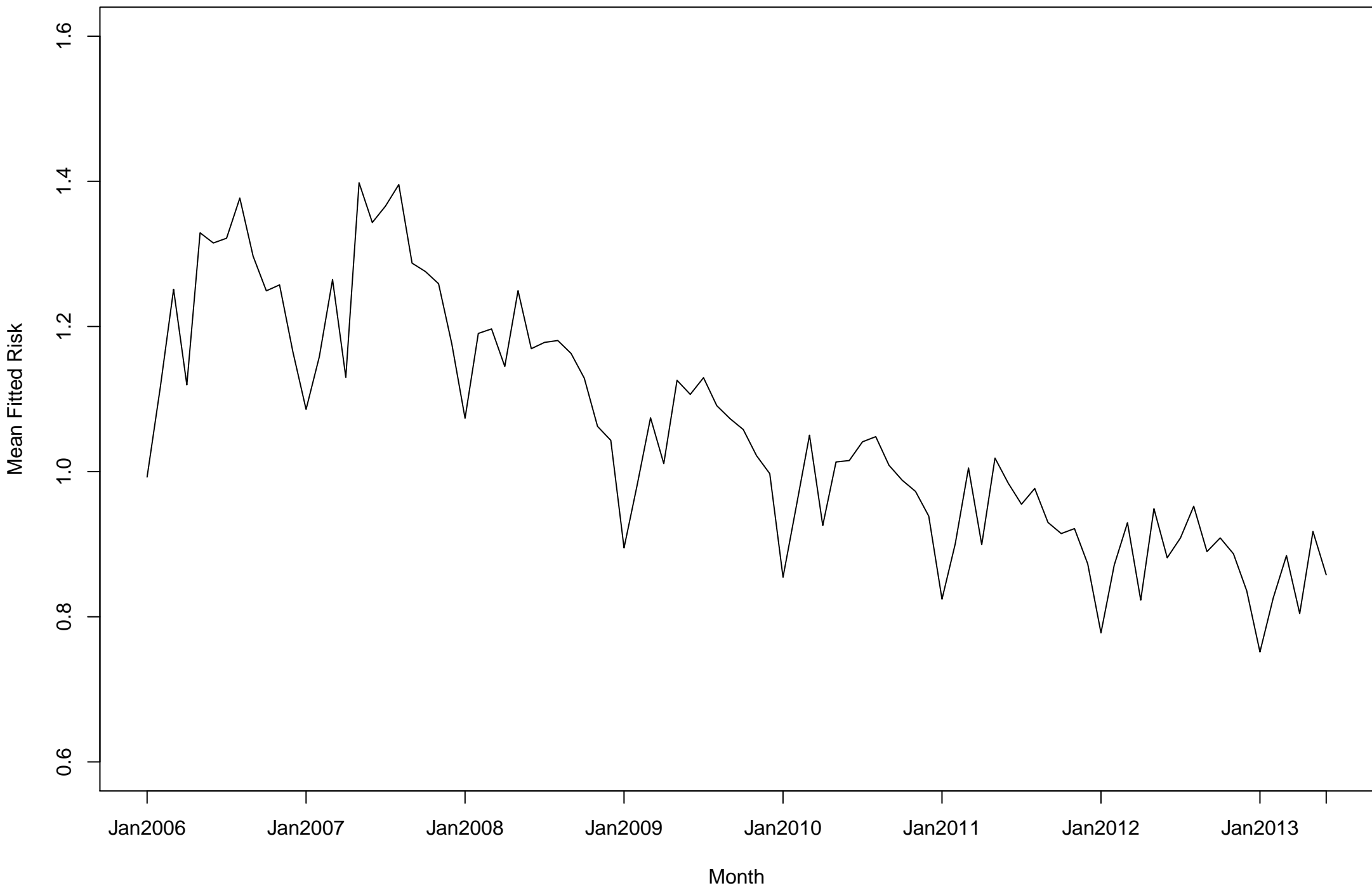

Supplement: Supplementary file 1 [file ijerph-14-00146-s001.zip › Supplementary/Figures/Mod6Trend.pdf]

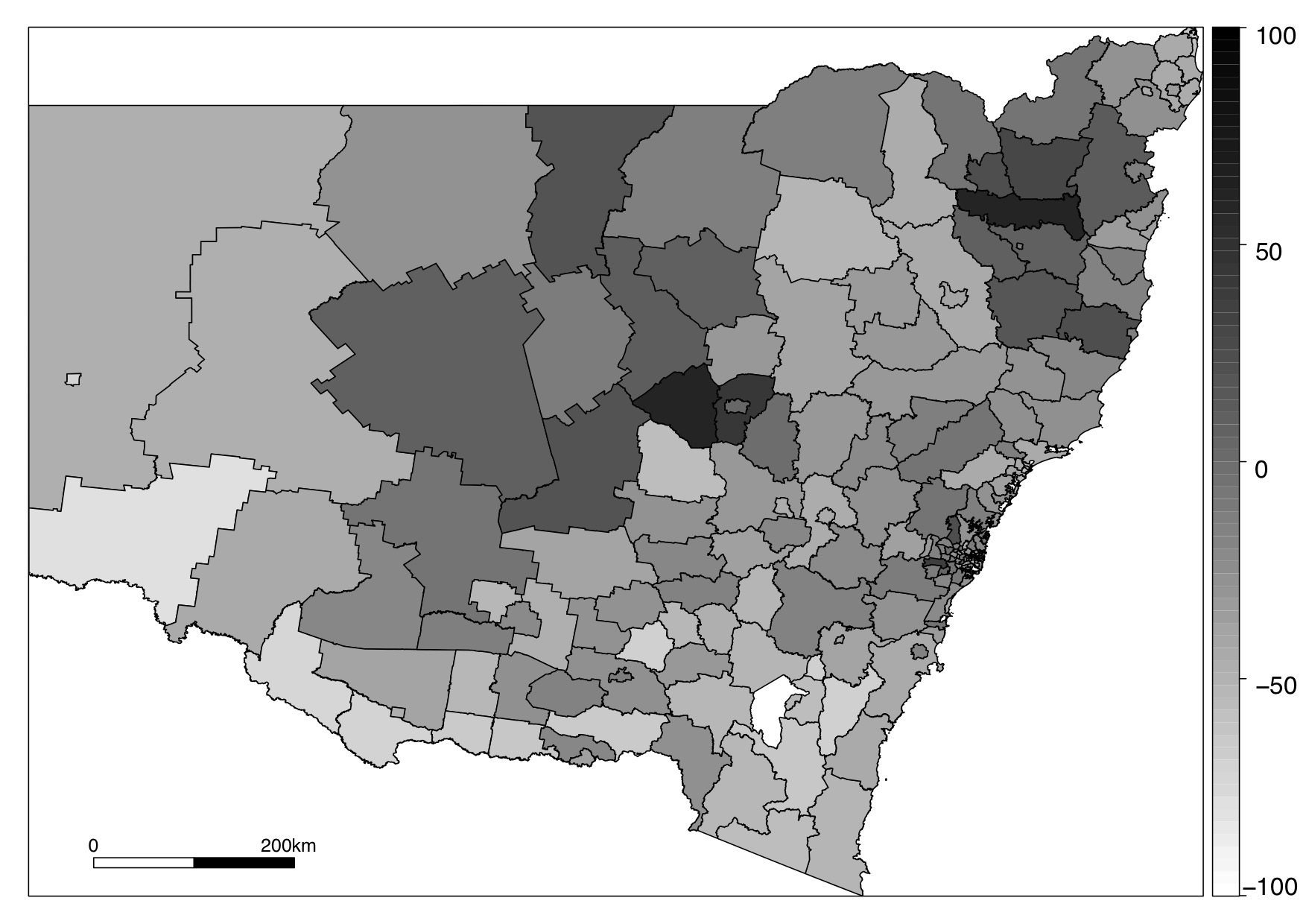

Supplement: Supplementary file 1 [file ijerph-14-00146-s001.zip › Supplementary/Figures/Mod7Change.jpg]

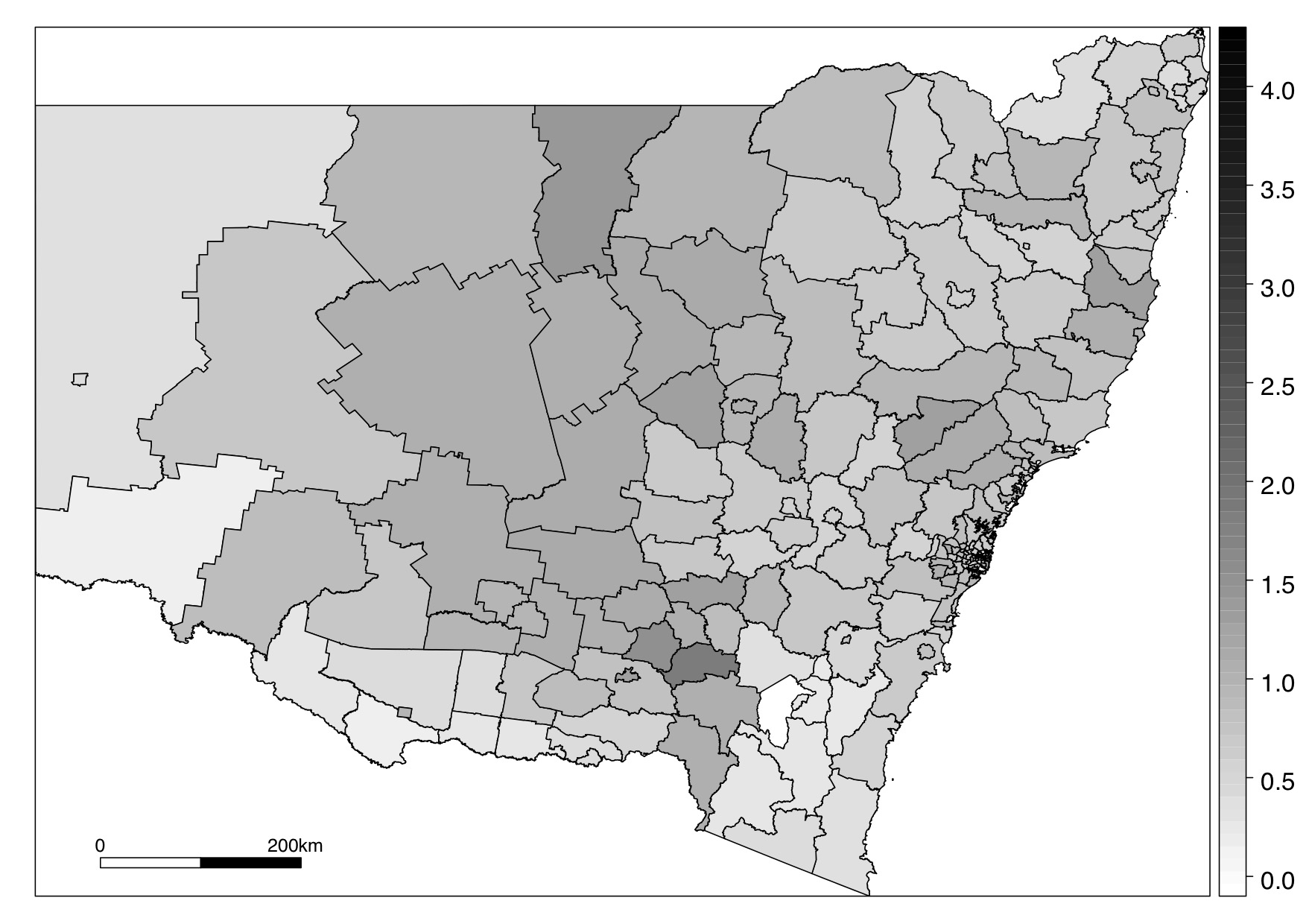

Supplement: Supplementary file 1 [file ijerph-14-00146-s001.zip › Supplementary/Figures/Mod7Final.jpg]

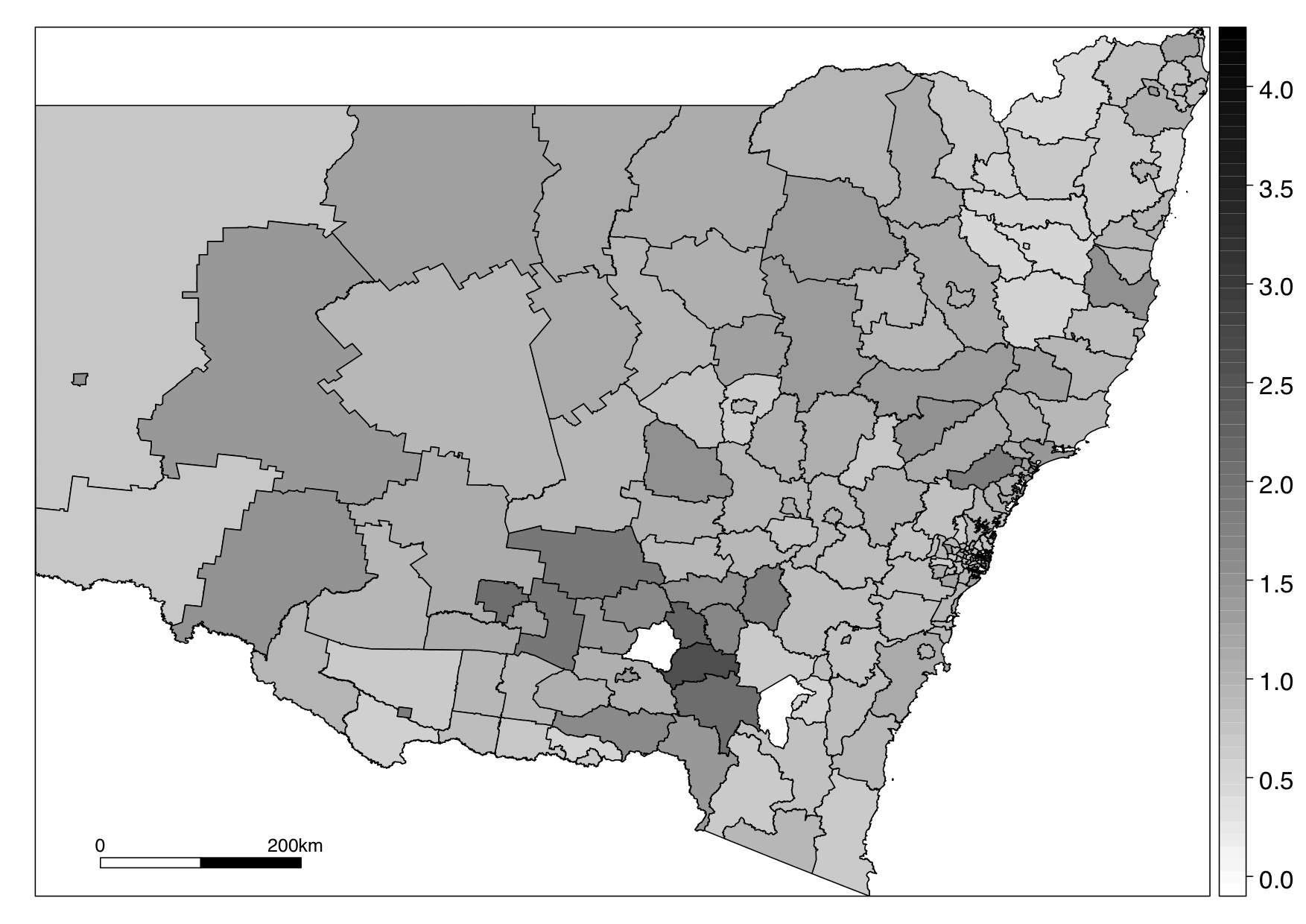

Supplement: Supplementary file 1 [file ijerph-14-00146-s001.zip › Supplementary/Figures/Mod7Start.jpg]

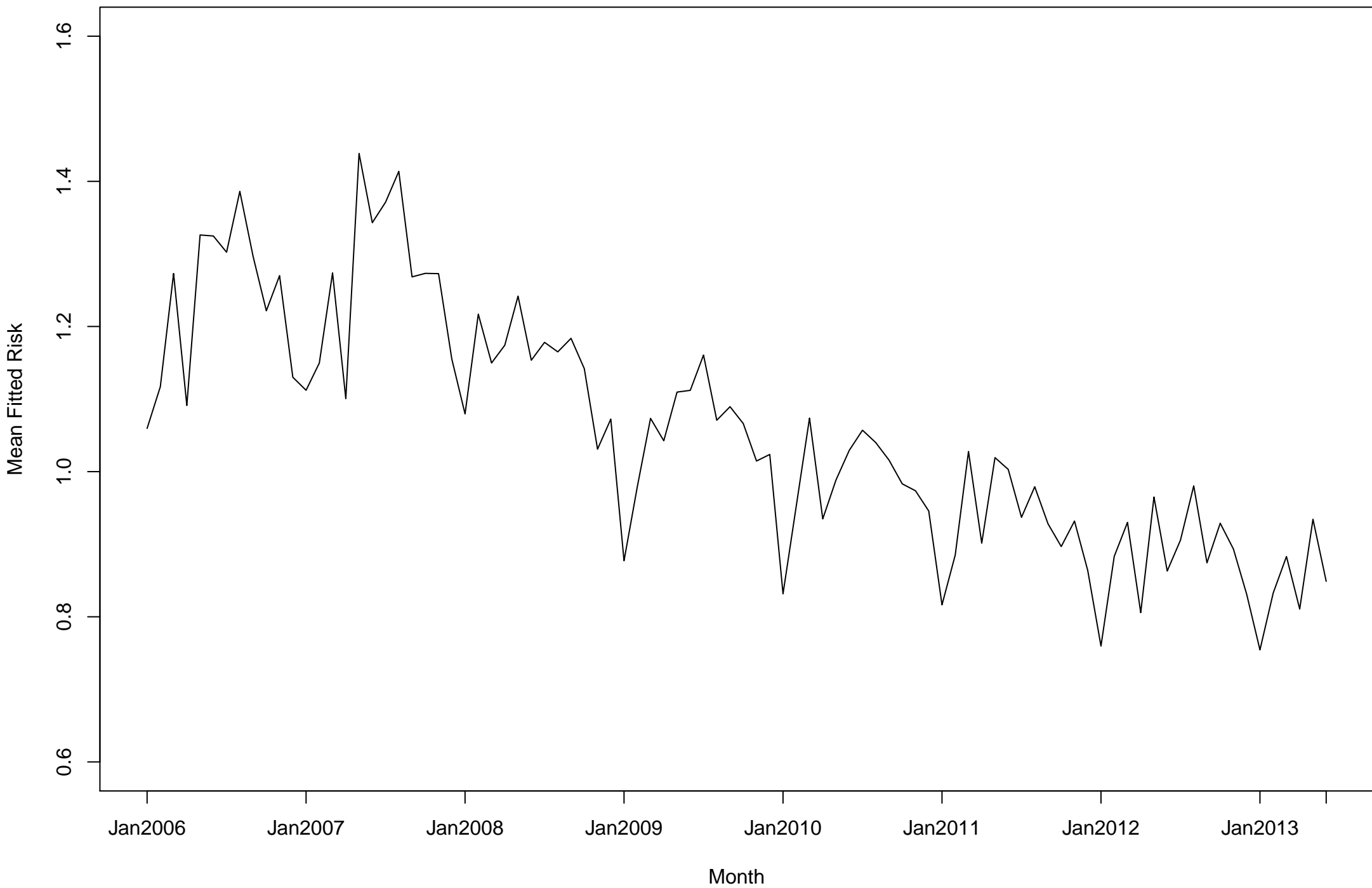

Supplement: Supplementary file 1 [file ijerph-14-00146-s001.zip › Supplementary/Figures/Mod7Trend.pdf]
